# Floating Carbon Nitride Composites for Practical Solar Reforming of Pre‐Treated Wastes to Hydrogen Gas

**DOI:** 10.1002/advs.202207314

**Published:** 2023-05-12

**Authors:** Stuart Linley, Erwin Reisner

**Affiliations:** ^1^ Yusuf Hamied Department of Chemistry University of Cambridge Lensfield Road Cambridge CB21EW UK

**Keywords:** biomass, floating catalyst, plastic, scaling, solar fuels, solar reforming

## Abstract

Solar reforming (SR) is a promising green‐energy technology that can use sunlight to mitigate biomass and plastic waste while producing hydrogen gas at ambient pressure and temperature. However, practical challenges, including photocatalyst lifetime, recyclability, and low production rates in turbid waste suspensions, limit SR's industrial potential. By immobilizing SR catalyst materials (carbon nitride/platinum; CN*_x_* |Pt and carbon nitride/nickel phosphide; CN*_x_* |Ni_2_P) on hollow glass microspheres (HGM), which act as floating supports enabling practical composite recycling, such limitations can be overcome. Substrates derived from plastic and biomass, including poly(ethylene terephthalate) (PET) and cellulose, are reformed by floating SR composites, which are reused for up to ten consecutive cycles under realistic, vertical simulated solar irradiation (AM1.5G), reaching activities of 1333 ± 240 µmol_H2_ m^−2^ h^−1^ on pre‐treated PET. Floating SR composites are also advantageous in realistic waste where turbidity prevents light absorption by non‐floating catalyst powders, achieving 338.1 ± 1.1 µmol_H2_ m^−2^ h^−1^ using floating CN*_x_* versus non‐detectable H_2_ production with non‐floating CN*_x_* and a pre‐treated PET bottle as substrate. Low Pt loadings (0.033 ± 0.0013% m/m) demonstrate consistent performance and recyclability, allowing efficient use of precious metals for SR hydrogen production from waste substrates at large areal scale (217 cm^2^), taking an important step toward practical SR implementation.

## Graphical abstract

Reusable floating composites of carbon nitride containing hollow glass microspheres separate to the air‐water interface where they catalyze solar reforming (SR) reactions, transforming waste streams to hydrogen gas. Driven by sunlight, the floating composite produces hydrogen gas from plastic and biomass substrates over consecutive cycles and enables SR in turbid waste suspensions, addressing some key concerns for potential application.

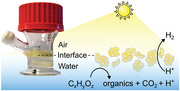

## 1 Introduction

Recycling of waste plastic and the production of CO_2_‐neutral fuels are two critical contemporary environmental challenges. Currently, recycling is suboptimally implemented with an estimated global recycling rate of only 9% and less than 1% of plastics being recycled more than once.^[^ ^1^, ^2^ ^]^ Hydrogen, often considered as the frontrunning energy vector to replace carbon‐rich fossil fuels,^[^ ^3^ ^]^ is most commonly produced using “grey” (CO_2_‐releasing) methods, accounting for almost the entirety of global production and releasing ≈900 Mt_CO2_ in 2020.^[^ ^4^ ^]^ Solar reforming (SR) offers a potential pathway to circular carbon use and clean production of hydrogen through dual functional photocatalysis that makes use of both oxidation and reduction half‐reactions, a critical requirement for an efficient heterogeneous photocatalytic process.^[^ ^5^, ^6^ ^]^ The oxidation half‐reaction has the potential to valorize diverse waste streams, including plastics and biomass, into useful materials, while the reduction half‐reaction offers a “green” alternative to “grey” hydrogen. Other, more conventional technologies, such as gasification (partial thermal oxidation to produce syngas), liquefaction (direct biomass conversion using higher pressure H_2_), or pyrolysis (thermal conversion in the absence of O_2_), also seek to use waste as feedstock materials to generate fuel and other useful chemicals, but often require high temperatures and pressures and provide poor product purity, restricting economic viability.^[^ ^6^, ^7^ ^]^ SR distinguishes itself through critical advantages such as its ability to be performed at ambient temperatures (20–60 °C) and the possibility of controlled catalytic oxidation for selective product synthesis.^[^ ^7^, ^8^ ^]^ In this way, SR can be more easily implemented and help mitigate plastic waste or use abundant waste biomass while contributing to an energy transition using H_2_ as energy carrier.

SR starts with a photocatalyst that absorbs solar energy, promoting electrons from the valence to the conduction band. Those photoexcited conduction band electrons are then transferred to a hydrogen evolution co‐catalyst (e.g., Pt, Ni_2_P) and the valence band holes directly oxidize organic substrates, such as soluble plastic or biomass monomers, thereby replenishing the electrons and providing protons for reduction.^[^ ^6^ ^]^ The theoretical overall reaction coupling the complete oxidation of organics to CO_2_ and reduction of water to H_2_ is described by Equation (1)

(1) $$C_{x}H_{y}O_{z}+\left(2x-z\right)H_{2}O\overset{h\nu }{\to }\left(2x-z+\frac{y}{2}\right)H_{2}+xCO_{2}$$

SR also offers a thermodynamic advantage over photocatalytic water splitting by oxidizing organic substrates, such as ethylene glycol (EG) (a monomeric component of polyethylene terephthalate [PET]; C_2_H_6_O_2_ + 2H_2_O → 5H_2_ + 2CO_2_, ΔG° = 14 kJ mol^−1^) or glucose (a monomeric component of cellulose; C_6_H_12_O_6_ + 6H_2_O → 6CO_2_ + 12H_2_, ΔG° = −35 kJ mol^−1^), giving a reforming reaction (*E* ^0^ = ≈0.0 V vs reversible hydrogen electrode [RHE])^[^ ^9^ ^]^ at approximately the H_2_ evolution potential, as opposed to water oxidation which has a high thermodynamic (H_2_O → H_2_ + ½O_2_, Δ*G*° = 237 kJ mol^−1^; *E* ^0^ = 1.23 V vs RHE) and kinetic energy barrier.^[^ ^7^ ^]^ While, in principle, SR produces CO_2_ as an end oxidation product, in practice, the reaction does not proceed to completion and >50% of the carbon remains as small organic products (e.g., formate) dissolved in solution with the remaining mineralized CO_2_ fixed as CO_3_^2−^ or HCO_3_^−^ under alkaline conditions, allowing SR to sequester carbon and concentrate CO_2_ for future processing.^[^ ^10^ ^]^

Photoreforming was first demonstrated using a UV‐light absorbing RuO_2_/TiO_2_/Pt photocatalyst in a sugar, starch, or cellulose substrate solution,^[^ ^11^ ^]^ with a more recent focus on using solar light and application to a wider range of realistic waste materials, including lignocellulose,^[^ ^12^, ^13^ ^]^ polymers such as PET, polylactic acid, and polyurethane,^[^ ^9^, ^14^ ^]^ and food waste.^[^ ^15^ ^]^ Further materials development has focused on utilizing visible light‐absorbing photocatalysts, such as CdS/CdO*_x_*,^[^ ^14^ ^]^ carbon nitride (CN*_x_*),^[^ ^9^, ^12^, ^16^ ^]^ or carbon dots,^[^ ^17^ ^]^ and inexpensive co‐catalysts as an alternative to noble metals such as Ni_2_P,^[^ ^9^ ^]^ MoS_2_,^[^ ^13^ ^]^ and NiO.^[^ ^18^ ^]^ Currently, waste solar reforming offers H_2_ production with a smaller carbon footprint than standard steam methane reforming, but a variety of improvements need to be made to achieve cost‐competitiveness and market‐readiness such as optimized photocatalyst durability, reuse, and application on a variety of real waste materials and real wastewaters.^[^ ^10^, ^19^, ^20^ ^]^ Among these improvements, photocatalyst durability is a critical consideration, with catalyst reuse lifetimes of at least 1 year proposed as necessary for cost effective H_2_ production.^[^ ^10^ ^]^

CN*_x_* recycling has been deployed using three methods: magnetization, immobilization, and as photocatalytic membranes.^[^ ^21^ ^]^ In the former two, CN*_x_* is directly exposed to the substrate solution for direct oxidation and reduction, whereas in the latter, the substrates are first removed from the solution by adsorption, then subsequently oxidized by CN*_x_* photocatalysis.^[^ ^21^ ^]^ In general, immobilization techniques and membranes are preferred over magnetic recovery due to lower maintenance, stability, and ease in efficient recovery from remaining waste solutions or solids. Membranes also present challenges such as fouling, oxidation damage from the photocatalyst, and scaling challenges due to their complicated production processes.^[^ ^21^ ^]^

Catalyst immobilization can be performed on a variety of support materials ranging from static, macroscopic supports, such as glass plates, tubes, or reactor vessel walls, to free supports such as glass beads, activated carbon, or sand.^[^ ^22^, ^23^ ^]^ For example, large‐scale reactors using immobilized photocatalysts have demonstrated consistent H_2_ production over months with solar‐to‐hydrogen efficiencies of up to 0.76% (SrTiO_3_:Al water splitting)^[^ ^24^, ^25^ ^]^ and 0.12% (mesoporous CN*_x_* with a sacrificial electron donor).^[^ ^26^ ^]^ In the context of SR, CN*_x_* has already been immobilized on flat glass panels as part of a flow‐through reactor, reaching activities of 52 ± 3 µmol m^−2^ h^−1^ with a Ni_2_P cocatalyst and pre‐treated PET substrate in 0.5 mol L^−1^ KOH.^[^ ^16^ ^]^ Though significant recent progress has been made on photocatalytic panel systems, slurry‐based systems may present a more scalable and economic approach enabled by improved light harvesting and mass transfer, allowing higher photon conversion efficiency.^[^ ^27^ ^]^ Among free supports suitable for application in a slurry system, floating materials have gained attention due to their enhanced light harvesting, gas exchange, and recovery resulting from catalyst localization at the surface of the solution.^[^ ^23^, ^28^, ^29^ ^]^ Already explored for water treatment, floating composites make use of robust, low‐density support materials such as expanded perlite,^[^ ^30^, ^31^ ^]^ fly ash cenospheres,^[^ ^32^ ^]^ hollow glass microspheres (HGMs),^[^ ^29^ ^]^ and polymer beads.^[^ ^33^ ^]^ The integration of fuel‐generating catalysts with floating platforms allows for versatile and decentralized deployment scenarios such as efficient use with turbid waste (TW) streams or enabling fuel production over open waters.^[^ ^34^ ^]^ To date, the scope of SR with reusable photocatalyst composites has been limited to panel immobilization and exploring floating SR composites is a promising next step for further scaling this technology.

**Figure 1:**
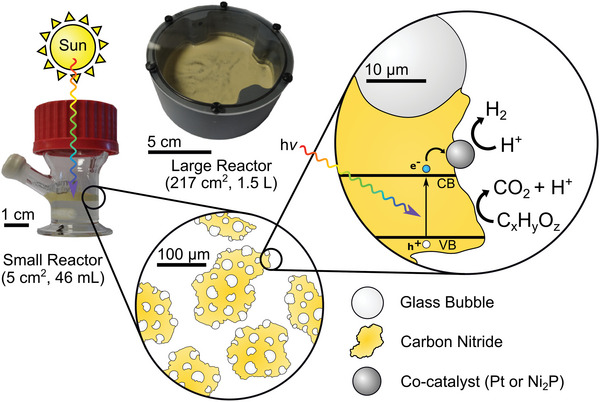
Floating solar reforming catalyst composite structure and mechanism. The floating composite (1 cm scale, in a small reactor with a surface area of 5 cm^2^ and volume of 46 mL) gravimetrically separates to the surface of an aqueous system. The structure of the floating composite (100 µm scale) comprises continuous CN*_x_* particles with interspersed glass bubbles. A hydrogen evolution co‐catalyst (Ni_2_P or Pt; 10 µm scale) facilitates the reduction of protons to H_2_ gas while organics oxidation is performed over CN*_x_*. The floating composite was also applied at a larger scale in a large reactor (5 cm scale) with a surface area of 217 cm^2^ and volume of 1.5 L.

Here, we introduce the deposition of CN*_x_* on low‐density HGMs, which enable floating in aqueous media, and couple this composite with a benchmark noble metal H_2_ evolution co‐catalyst (platinum; HGM/CN*_x_* |Pt), as well as noble‐metal‐free nickel phosphide (HGM/CN*_x_* |Ni_2_P) for SR (Figure 1). We demonstrate that such floating SR composites can be easily recovered and reused over ten consecutive SR trials using realistic illumination conditions and pre‐treated model wastes including pre‐treated biomass (cellulose) and plastic (PET). We also track the change in SR activity in both co‐catalyst systems and show the advantage of floating materials in turbid waste (TW) environments in a large areal‐scale SR system using real and model wastes. Floating photocatalyst composites offer a practical method for scaling SR without loss in areal activity in a solar‐light‐driven process.

## 2 Results and Discussion

### 2.1 Synthesis of Floating Carbon Nitride

Several candidate floating support materials, including two varieties of perlite and five varieties of HGMs with different crush strengths, particle diameters, and densities, were screened for floating and durability, and iM30k (3M Company; 18 µm average particle diameter) was selected for all experiments going forward as it had the highest floating mass fraction following washing procedures (Table S1, Supporting Information). In addition to CN*_x_*, P25 TiO_2_, and nitrogen‐doped graphitic carbon dots^[^ ^17^ ^]^ were also assessed as potential SR photocatalysts anchored to the HGMs using a sodium metasilicate‐based binder, but neither was selected for further study due to poor composite stability and co‐catalyst incompatibility (see Supporting Information for additional details; “alternative composite compositions”).

Floating CN*_x_* composites were prepared by pyrolysis of melamine in the presence of HGMs at a temperature of 550 °C. The resulting yellow cake was then extracted from the crucible, milled with a mortar and pestle, and washed by gravimetric separation in a separatory funnel to isolate the floating fraction of the composite. Synthesis conditions varying the amount of melamine (2–8 g) while maintaining a constant HGM content (2 g) were tested to assess how the yield and CN*_x_* content of the floating composite changed (**Table** **1**). Decreasing the HGM:melamine ratio introduced a composite yield versus quality trade‐off whereby loading the HGMs with more CN*_x_* resulted in a smaller fraction of the product with a density low enough to float on water. By using a 1:1 ratio of HGM:melamine, 50% of the final product was a useable floating composite with a CN*_x_* content of 32%, while using a 1:4 ratio of HGM:melamine produced a floating fraction of only 12% in the final product, but with a 43% CN*_x_* content (**Figure** **1**).

**Table 1:** Mass, floating fraction, and carbon nitride content of HGM/CN*_x_* samples prepared by pyrolysis of melamine (2–8 g) in the presence of hollow glass microspheres (HGM; 2 g)

Sample mass ratio HGM:melamine	*m*_synthesis_ [g]^a)^	*m*_float_ [g]^b)^	Floating fraction [%]	CN*_x_* fraction [%]^c)^
1:1	2.4529	1.2267	50	32.3
1:2	3.8404	0.9845	25.6	39.5
1:3	5.2202	0.8576	16.4	44.5
1:4	6.3154	0.7684	12.2	43.3

^a)^ Mass of iM30k/CN*_x_* composite recovered from pyrolysis;

^b)^ Mass of iM30k/CN*_x_* composite recovered after washing 3× with water by floating until the solution was clear;

^c)^ CN*_x_* mass fraction of floating composite from CHN combustion microanalysis.

**Figure 2:**
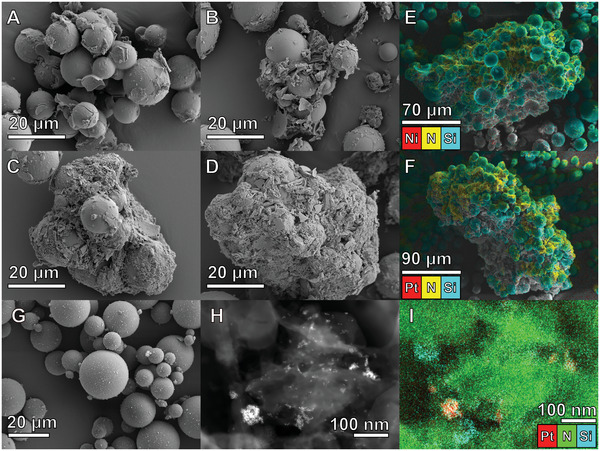
SEM images showing the morphology of floating CN*_x_* composites differentiated by their HGM:melamine ratio. A) 1:1, B) 1:2, C) 1:3, D) 1:4 (scale bar shared by panels A–D), E) colored EDX overlay showing Ni, N, and Si of 1:3 with Ni_2_P co‐catalyst, F) colored EDX overlay showing Pt, N, and Si of 1:3 with Pt co‐catalyst, G) iM30k glass bubbles, H) STEM‐HAADF of HGM/CN*_x_* |Pt (1:3) showing elemental contrast, I) EDX overlay on STEM‐HAADF of HGM/CN*_x_* |Pt (1:3) showing Pt, N, and Si.

The HGM:melamine ratio also influenced the size and morphology of the floating composite (**Figure** **2**). At an HGM:melamine ratio of 1:1, the composite appeared to be primarily individual HGMs (Figure 2G) coated with a thin shell or small particles of CN*_x_* (Figure 2A). As the mass of melamine was increased with respect to the mass of HGMs, the CN*_x_* mass‐fraction of the composite also increased, and at a HGM:melamine ratio of 1:3 or above, the composite morphology became continuous clusters of CN*_x_* with embedded HGMs (Figure 2C,D). The elemental distribution of N and Si from EDS elemental mapping in Figure 2E,F supports this interpretation, demonstrating high N content throughout the rough, continuous phase (yellow; CN*_x_*) while the spherical HGM particles are evidenced by their high Si content (blue; SiO_2_). The energy dispersive X‐ray (EDX) maps in Figure 2 show floating CN*_x_* composites prepared from an HGM:melamine ratio of 1:3 with deposited Ni_2_P (Figure 2E) or Pt (Figure 2F), but the co‐catalyst elemental content is low enough (≈0.9% Ni; ≈0.1% Pt) that it is not easily discernable in the overlay mapping. Individual elemental maps can be found in the Supporting Information (Figures S4 and S5, Supporting Information). ICP‐OES characterization of the composite confirmed the low Ni and Pt loadings, with an average Ni loading in HGM/CN*_x_* |Ni_2_P (1:3 HGM:melamine) of (1.26 ± 0.07)% m/m and a Pt loading in HGM/CN*_x_* |Pt (1:3 HGM:melamine) of (0.033 ± 0.0013)% m/m. Though the Pt loading is remarkably low, its presence on the surface of the composite was confirmed with scanning transmission electron microscopy with high angle annular dark field (STEM/HAADF) and EDX where the Pt appeared as nano‐scale clusters ranging from 5 to 50 nm (Figure 2H,I and Figure S6, Supporting Information).

The HGM/CN*_x_* composites showed remission spectra characteristic of CN*_x_* with absorbance band edges at ≈450 nm, though all HGM/CN*_x_* composites showed higher remission using diffuse‐reflectance UV/vis spectroscopy than CN*_x_* in the <400 nm range. The addition of Pt or Ni_2_P co‐catalysts to the HGM/CN*_x_* (1:3 HGM:melamine) composite was also evidenced by a color change (HGM/CN*_x_*: yellow; HGM/CN*_x_* |Ni_2_P: grey; HGM/CN*_x_* |Pt: grey‐yellow) and increased remission (Figure S7, Supporting Information). These changes in color and reflectance likely result from the introduction of continuous energy bands in the Pt or Ni_2_P co‐catalysts, which function as electron collectors and promote the proton reduction half‐reaction, and therefore do not contribute to photo‐charge generation.^[^ ^35^ ^]^

### 2.2 Solar Reforming Performance of Floating Carbon Nitride

**Figure 3:**
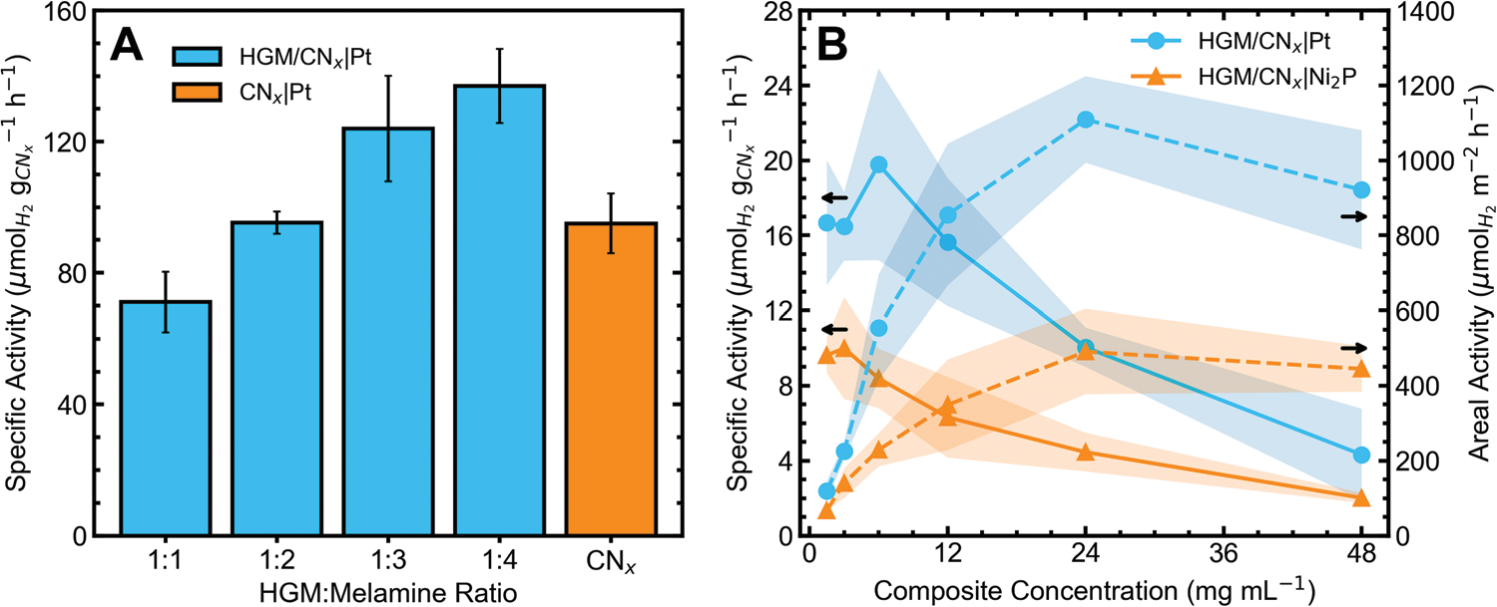
Solar reforming activity of floating CN*_x_* composites using ethylene glycol (EG) as a substrate. A) Activity of composites produced from different HGM:melamine ratios using horizontal illumination in small vials (100 mW cm^−2^, *V* = 2 mL, 1 mol L^−1^ KOH, [EG] = 25 mg mL^−1^, [CN*_x_*] = 1.5 mg mL^−1^, stirred, *T* = 25 °C), B) Activity of 1:3 HGM:melamine ratio HGM/CN*_x_* composite at different concentrations under small area (4.9 cm^2^) vertical irradiation (100 mW cm^−2^, *V* = 5 mL, 1 mol L^−1^ KOH, [EG] = 25 mg mL^−1^, no stirring, *T* ≈ 45 °C). Solid lines show specific activity (µmol_H2_ g_CNx_^−1^ h^−1^) and dashed lines show areal activity (µmol_H2_ m^−2^ h^−1^). The shaded area surrounding each line shows the standard deviation of each triplicate.

The various HGM/CN*_x_* composites prepared with different HGM:melamine ratios were assessed for their activity in small‐vial (7.9 mL) SR trials. An amount of HGM/CN*_x_* was added to each vial based on the CN*_x_* content of the composite (Table 1) such that the final concentration of CN*_x_* in each experiment was 1.5 mg mL^−1^. A substrate solution containing 1 mol L^−1^ KOH and 25 mg mL^−1^ EG was added to each vial containing HGM/CN_x_ (experiments) or CN_x_ (control), along with 1.6 µL H_2_PtCl_6_ (8 wt%) solution as an in situ photodeposited co‐catalyst. Horizontal irradiation with a stirred catalyst system was used to allow a direct comparison between the SR activity of the floating composites with non‐floating CN*_x_*, as well as a comparison with previous literature using similar experimental setups.^[^ ^9^, ^14^, ^15^, ^16^ ^]^ Despite normalizing the amount of CN*_x_* in each vial, the specific (mass‐normalized) activity of the different HGM/CN*_x_* samples generally increased as the HGM:melamine synthesis ratio decreased. The 1:4 HGM:melamine sample demonstrated specific activity comparable to the 1:3 HGM:melamine sample and both samples exceeded the activity of CN*_x_* with photo‐deposited Pt (**Figure** **3**A and Table S2, Supporting Information), showing that the incorporation of CN*_x_* into a floating composite does not incur a trade‐off between floating and catalytic activity when relying on a Pt co‐catalyst photo‐deposited from H_2_PtCl_6_.

Exclusion control experiments demonstrated no SR activity in the absence of the co‐catalyst or substrate. SR activity was highest over chemically deposited Pt on CN*_x_* (CN*_x_* |Pt_chem_), which displayed the highest Pt loading (0.91 ± 0.017 wt%) with 25–40% lower activity at lower Pt loadings (CN*_x_* |Pt_photo_: 0.19 ± 0.058 wt%, HGM/CN*_x_* |Pt_photo_: 0.13 ± 0.018 wt%, HGM/CN*_x_* |Pt_chem_: 0.033 ± 0.0013 wt%; Table S3, Supporting Information). SR activity may also be affected by the type of deposition, in addition to the Pt surface loading, with chemical reduction producing stable metallic deposits that are not deactivated over time, as in photo‐deposited Pt, possibly contributing to higher sustained activities.^[^ ^36^ ^]^ Based on its specific activity and floating fraction recovered after synthesis, the 1:3 HGM:melamine floating composite was selected as the HGM/CN*_x_* material used in all further SR experiments.

The HGM/CN*_x_* composites were then applied in vertically irradiated SR conditions using small glass reactors (SA = 4.9 cm^2^ *V* = 46 mL; Figure S2, Supporting Information) at different composite loadings with both Ni_2_P and Pt co‐catalysts (chemically deposited) to determine appropriate conditions for scaled‐up catalyst reuse trials (Figure 3B and Table S4, Supporting Information). As the HGM/CN*_x_* (1:3 HGM:melamine) concentration increased from 1.5 (1.53 mg cm^−2^) to 48 mg mL^−1^ (49 mg cm^−2^), the specific activity of the composite declined from a maximum of 19.8 to 4.9 µmol_H2_ g_CNx_^−1^ h^−1^ with a Pt co‐catalyst and 10 to 2.2 µmol_H2_ g_CNx_^−1^ h^−1^ with a Ni_2_P co‐catalyst. The areal activity, which reports the time‐normalized amount of H_2_ produced per area, regardless of the amount of composite in the reaction, was found to increase with composite loading from 119 to 1109 µmol_H2_ m^−2^ h^−1^ at 1.5 and 24 mg mL^−1^, respectively, with a Pt co‐catalyst and from 68 to 490 µmol_H2_ m^−2^ h^−1^ at 1.5 and 24 mg mL^−1^, respectively, with a Ni_2_P co‐catalyst. This highlights the trade‐off between the efficient use of photocatalyst material and maximum hydrogen production. By using a high concentration of floating composite, high areal activities can be achieved, but after a composite concentration of 12 mg mL^−1^, the benefit of adding an additional catalyst is small. For example, in the case of HGM/CN*_x_* |Pt, doubling the composite concentration from 3 to 6 mg mL^−1^ increases the H_2_ yield by a factor of 2.5, while doubling again from 6 to 12 mg mL^−1^ only increases yield by a factor of 1.5. Based on these findings, a composite loading of 12 mg mL^−1^ was selected for further trials to allow for efficient use of material and balance specific and areal activities.

The mechanism of SR over HGM/CN*_x_* |Pt and HGM/CN*_x_* |Ni_2_P is expected to proceed according to SR over CN*_x_* |Pt or CN*_x_* |Ni_2_P in the absence of the inert silica support material as described in previous work.^[^ ^9^ ^]^ Briefly, photo‐generated electrons and holes drive proton reduction and substrate oxidation, respectively, with the Pt or Ni_2_P co‐catalyst serving as an electron collector and hydrogen evolution co‐catalyst. Substrate oxidation proceeds non‐selectively through direct electron transfer to CN*_x_* rather than an OH· mediated pathway and produces a wide variety of intermediate products with a final mineralized product of CO_3_^2−^ in alkaline media.^[^ ^9^ ^]^ Though the mechanism should remain the same, CN*_x_* or HGM/CN*_x_* light absorbers may be affected by altered exposure to light and decreased kinetics, likely from reduced mass transfer between the substrate and the photocatalyst in the absence of stirring, particularly if the catalyst is not uniformly distributed across the solution surface in a floating system. In this study, a lower specific activity was therefore observed under such conditions (Figure 3B). Evaluating the effect of light orientation on photocatalytic activity is challenging as gravity remains a factor that affects heterogeneous catalyst distribution, particularly for floating systems, and liquid geometry. Nevertheless, it is anticipated that the overall effect of light orientation would be minimal.

### 2.3 Recyclability of Floating Carbon Nitride

**Figure 4:**
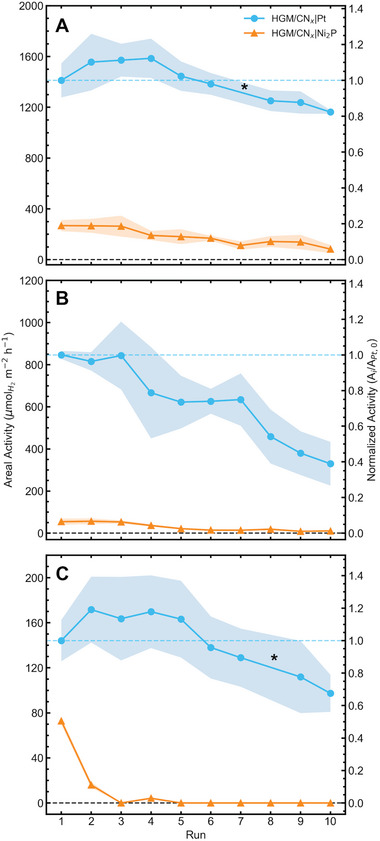
Small area (SA = 4.9 cm^2^) solar reforming areal activity of HGM/CN*_x_* |Pt and HGM/CN*_x_* |Ni_2_P composites using top‐down irradiation (100 mW cm^−2^, *V* = 5 mL, 1 mol L^−1^ KOH, [substrate] = 25 mg mL^−1^, no stirring, 2 h, T ≈ 45 °C) over ten consecutive trials with different substrates: A) ethylene glycol, B) polyethylene terephthalate, and C) cellulose. The shaded area surrounding each line shows the standard deviation from each triplicate. * show where outlying data points have been removed from the figure (Figure S9, Supporting Information).

The recyclability of the HGM/CN*_x_* composites was assessed in the same small reactors (surface area [SA] = 4.9 cm^2^ *V* = 46 mL) under vertical illumination (AM1.5G) using three different substrates: EG, PET, and cellulose. The EG and PET solutions were prepared in 1 mol L^−1^ KOH, reflecting the pre‐treatment conditions for solid PET, while the cellulose was prepared by enzymatic pre‐treatment and had a pH 5 solution containing 50 mmol L^−1^ sodium acetate. The same HGM/CN*_x_* composite (1:3 HGM:melamine) was applied in ten consecutive 2 h SR trials and each set of ten runs was performed in triplicate using different HGM/CN*_x_* samples. The floating HGM/CN*_x_* mass recovery after ten trials averaged (58.9 ± 6.4)% in the small reactors with no significant difference in recovery between the HGM/CN*_x_* |Pt (61.4 ± 3.7%) and HGM/CN*_x_* |Ni_2_P (56.5 ± 8.3%) samples (Table S5, Supporting Information). Separation was rapid with the visual clarity of the aqueous phase achieved over 10 min of separation time (Figure S8, Supporting Information). The SR activity of the HGM/CN*_x_* |Pt was found to decrease by a small degree with each cycle in each substrate solution (**Figure** **4** and Table S6, Supporting Information), reaching a final areal activity of (82.3 ± 1.0)% in EG (Figure 4A), (31.1 ± 12.1)% in PET (Figure 4B), and (67.5 ± 11.4)% in cellulose (Figure 4C). Except for PET, the percent decrease in activity approximates the percent mass loss of the HGM/CN*_x_* composite. Compared with a non‐floating composite (CN*_x_*), the HGM/CN*_x_* showed much greater reuse. The non‐floating composite was unable to be separated from the reforming solution without assistance (e.g. filtration, centrifugation), resulting in significant catalyst loss and a decrease in activity between experiments (Figure S10, Supporting Information). Overall, the consistent activity of the HGM/CN*_x_* |Pt samples points to the CN*_x_* |Pt system as capable of maintaining SR performance over multiple trials, with loss in activity attributable to loss of the composite material itself (either through aspiration between trials or breakage and sinking of the composite). In the small reactor trials, a meniscus effect in the 2.5 cm diameter cylindrical reactor caused the floating composite to move into a ring formation in the absence of stirring, clinging to the walls of the reactor over the 2 h irradiation period, and the speed of this separation may have affected the H_2_ yield and may have been affected by solution characteristics such as surface tension.

The HGM/CN*_x_* |Ni_2_P composites showed a substantial decrease in areal activity over the ten cycles, reaching minimum activities of (31.1 ± 12.2)% in EG (Figure 4A), (19.6 ± 1.2)% in PET (Figure 4B), and complete loss of activity (0%; H_2_ not detected) in cellulose (Figure 4C). The individual plots of HGM/CN*_x_* |Ni_2_P areal activity in the small reactor recyclability trials can be found in the Supporting Information (Figure S11, Supporting Information) with scales that more clearly show the decline in activity from run to run. The decrease in activity exceeds the composite mass loss over ten cycles, pointing to a decline in the SR activity of the Ni_2_P cocatalyst. This is especially evident in the cellulose substrate solution (Figure 4C) where the activity rapidly drops to 0 over only three runs. This is likely due to the dissolution of the Ni_2_P co‐catalyst which has poor stability in acidic environments such as the pH 5 pre‐treated cellulose solution.^[^ ^37^ ^]^ Measuring the Ni content of the composite by ICP‐OES after ten cycles in the pre‐treated cellulose solution yielded (0.056 ± 0.005)% m/m compared to (1.56 ± 0.01)% after ten cycles in EG or pre‐treated PET solution, confirming the difference in stability between the different pH environments. Despite Ni still being present in a higher concentration than Pt in the HGM/CN*_x_* |Pt samples (0.03% m/m) the activity is far lower, and it is expected that this is a consequence of the degradation of the catalytically active Ni_2_P species. This result also highlights the versatility and robustness of the Pt cocatalyst as it can be applied in various pH environments and would therefore be applicable to a wider range of substrates requiring different pre‐treatment conditions.

**Figure 5:**
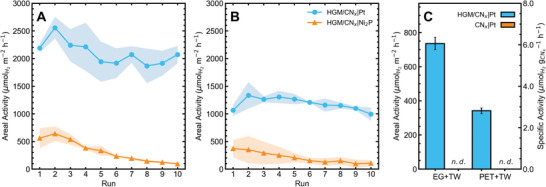
Large area (SA = 217.4 cm^2^) solar reforming areal activity of HGM/CN*_x_* |Pt and HGM/CN*_x_* |Ni_2_P composites using top‐down irradiation over ten consecutive trials (100 mW cm^−2^, 1 mol L^−1^ KOH, [substrate] = 25 mg mL^−1^, *V* = 217 mL, depth = 1 cm, no stirring, 2 h, *T* ≈ 45 °C) with different substrates: A) Ethylene glycol (EG), B) polyethylene terephthalate (PET), C) EG and PET (plastic bottle) in turbid waste (TW) solution. *n.d*. denotes “not detected.” The shaded area surrounding each line shows the standard deviation from each triplicate.

The performance of the HGM/CN*_x_* composites in a more realistic system mimicking real‐world conditions was assessed using a large poly(vinyl chloride) (PVC) reactor (217 cm^2^ 1.5 L) with a Plexiglas window to allow for vertical irradiation. The volumetric and areal concentrations of the floating composite from the small reactor experiments were maintained at 12 mg mL^−1^ and 12 mg cm^−2^ in this larger system with a fluid volume of 217 mL and depth of 1 cm. The areal activity HGM/CN*_x_* |Pt composite marginally declined over ten consecutive trials in both EG (**Figure** **5**A) and pre‐treated PET (Figure 5B), with activities reaching minimum values of (85.2 ± 14.3)% and (93.4 ± 11.4)% of the initial activity, respectively (Table S7, Supporting Information). The composite recovery was consistent across all triplicates over ten cycles, with an average of (68.2 ± 6.0)% (Table S5, Supporting Information). The HGM/CN*_x_* |Ni_2_P composite demonstrated a much more significant decline in activity, reaching minimum values of (31.1 ± 12.1)% in EG and (16.0 ± 2.9)% in PET (Figure 5A,B and Figure S12, Supporting Information). The Ni content in the composite was measured before and after ten solar reforming cycles to test whether loss of Ni_2_P might be causing the decline in activity, but it was found that the Ni content of the floating fraction increased by (0.31 ± 0.16) wt% (Figure S13, Supporting Information). The decrease in the activity of the HGM/CN*_x_* |Ni_2_P catalysts over multiple cycles in basic conditions is therefore not directly related to the dissolution or loss of Ni but may be due to surface modification of the Ni_2_P, for example, the formation of a passivating aerial oxidation layer that may block the catalytically active sites.^[^ ^38^ ^]^ This finding is also consistent with previous investigations of CN*_x_* |Ni_2_P that determined Ni content remains similar before and after photocatalysis, while activity and P content decreases significantly as Ni‐P and NiO*_x_* species are replaced with Ni(OH)_2_.^[^ ^9^ ^]^

Following ten re‐use cycles, the composite appeared to retain its original morphology. Large masses of CN*_x_* with embedded HGMs were evident throughout the sample and EDS qualitative analysis largely showed the same elemental distribution with Si and O localized in the spherical HGMs and C and N distributed in the interstitial material (Figures S14–S17, Supporting Information). Post‐catalysis, the composite also exhibited a strong potassium signal, likely residual K^+^ adsorbed to s‐triazine rings in the carbon nitride.^[^ ^39^ ^]^ The surface of the composite after ten re‐use cycles showed a slightly different appearance, with the carbon nitride appearing somewhat more connected and “web‐like” in comparison to the “before” images. Previous studies have reported the exfoliation and etching of carbon nitride under alkaline conditions (5 mol L^−1^ KOH) at mild temperatures (room temperature to 80 °C).^[^ ^39^ ^]^ Combustion microanalysis of the composites before and after the ten reuse cycles determined that the carbon nitride content of the composites was not substantially changed, with initial CN*_x_* contents of 46.6 ± 0.1% and 44 ± 0.1% for HGM/CN*_x_* |Pt and HGM/CN*_x_* |Ni_2_P, respectively, and final CN*_x_* contents of 44.6 ± 0.1% and 42.8 ± 0.1%. FTIR analysis of the composites before and after ten reuse cycles showed the appearance of a new shoulder peak at 970 cm^−1^ (Figure S18, Supporting Information) that could be attributed to the formation of amine oxide (N—O stretching) or epoxide (C—O—C stretching) groups through autooxidation of CN*_x_*.^[^ ^40^, ^41^ ^]^ Although the composite showed consistent morphology and stability upon repeated use in SR conditions, mechanical abrasion under stirred conditions resulted in substantial composite breakage with only 75–85% of the floating material recovered after 30–60 min of stirring (Table S8, Supporting Information) and substantial morphological change with many broken microspheres evident (Figure S19, Supporting Information). As long as the composite was not subjected to direct abrasion, it appeared stable and retained its activity and floating.

The areal activity of floating HGM/CN*_x_* composites under the SR conditions studied compares favorably to similar conditions previously explored for immobilized CN*_x_* on glass panels. The maximum activity of the HGM/CN*_x_* |Pt composite (217 cm^2^ 2 h, 1 mol L^−1^ KOH, 25 mg mL^−1^ EG, ≈5.4 mg_CNx_ cm^−2^) was observed to be 2558 ± 197 µmol_H2_ m^−2^ h^−1^, whereas previously reported CN*_x_* |Pt panels (1 cm^2^ 20 h, 0.5 mol L^−1^ KOH, 25 mg mL^−1^ EG, 1.92 mg_CNx_ cm^−2^) were found to exhibit 280 µmol_H2_ m^−2^ h^−1^ areal activity.^[^ ^16^ ^]^ Possible reasons for the increased activity in the floating system may include the increased surface density of CN*_x_*, greater surface area availability of CN*_x_* for SR reactions, or improved light harvesting. Initially, the floating HGM/CN*_x_* |Ni_2_P system also outperformed its panel‐immobilized counterpart with a maximum areal activity of 370 ± 158 µmol_H2_ m^−2^ h^−1^ (217 cm^2^ 2 h, 1 mol L^−1^ KOH, 25 mg mL^−1^ PET, ≈5.4 mg_CNx_ cm^−2^) compared to 52 ± 3 µmol_H2_ m^−2^ h^−1^ (25 cm^2^ 20 h, 0.5 mol L^−1^ KOH, 25 mg mL^−1^ PET, ≈1.92 mg_CNx_ cm^−2^), though the activity of the floating HGM/CN*_x_* |Ni_2_P composite was found to decline substantially over 20 h (ten consecutive cycles), while the panel system maintained steady activity over 20 h. This may be due to the nature of the recycling process which routinely exposes the catalyst to oxygen after every recycle to introduce fresh substrate solution, allowing a passivation layer to form, while the panels were operated continuously in an anoxic environment. Nevertheless, clear benefits in areal activity were realized in using the floating HGM/CN*_x_* system on free supports compared to CN*_x_* on static panel supports.

Scaling up the SR system had clear benefits in the case of HGM/CN*_x_* |Pt with increases of 728 ± 179 and 562 ± 152 µmol_H2_ m^−2^ h^−1^ over the SR system when using EG or PET, respectively. The benefits were less obvious when using HGM/CN*_x_* |Ni_2_P, but an increase of 180 ± 81 µmol_H2_ m^−2^ h^−1^ was observed using PET as a substrate. The increased areal activity may be due to a more consistent distribution of the floating catalyst across the large reactor surface. In the small reactors, surface tension effects and menisci caused floating particles to separate to the edges of the reactor, effectively decreasing the light‐harvesting area of the system. Two clear trends were established from the up‐scaled floating composite application: floating SR reactions can be scaled with an area without a loss in specific or areal activity, or composite recovery, and Pt is a stable co‐catalyst material suitable for application in multi‐use SR composites.

A significant benefit of the floating composite material was its SR capability in TW. To simulate a TW stream, mixed waste from a floatation separation process was pre‐treated in 1 mol L^−1^ KOH for 24 h at 80 °C and coarsely filtered through glass wool to produce a dark brown suspension which was then loaded with 25 mg mL^−1^ EG as a substrate (Figure S20, Supporting Information). In the absence of added substrate, the pre‐treated TW did not produce a detectable quantity of H_2_ over 24 h using HGM/CN*_x_* |Pt. Vertically irradiated SR experiments (in triplicates) using 217 mL (1 cm deep, 217 cm^2^ 2 h, 1 mol L^−1^ KOH, 25 mg mL^−1^ EG, 12 mg mL^−1^ composite) of the TW+EG solution in the large reactor demonstrated that non‐floating carbon nitride (CN*_x_* |Pt) was unable to produce a detectable quantity of H_2_ in a 2 h period while floating HGM/CN*_x_* |Pt produced 728.5 ± 2.7 µmol_H2_ m^−2^ h^−1^ under the same conditions (Figure 5C). Small‐vial control experiments in well‐mixed, side‐illumination conditions where the light exposure to both catalysts was equally demonstrated that the CN*_x_* |Pt catalyst had approximately the same performance as HGM/CN*_x_* |Pt in both clear (EG only) and TW conditions (Figure S21, Supporting Information). Realistic application of HGM/CN*_x_* |Pt was further demonstrated using pre‐treated PET from a plastic bottle in turbid waste showing similar results; the floating composite produced H_2_ at a rate of 338.1 ± 1.1 µmol_H2_ m^−2^ h^−1^ while the CN*_x_* |Pt catalyst produced no detectable H_2_ under vertical illumination. Under well‐mixed, horizontally illuminated conditions, the CN*_x_* |Pt produced H_2_ at a higher rate than HGM/CN*_x_* |Pt, demonstrating that the material is catalytically active when suitably exposed to simulated sunlight (Figure S21, Supporting Information). Thus, the floatability of the HGM/CN*_x_* |Pt composite can be an asset in static, vertically illuminated environments with non‐transparent waste streams more realistic to the practical application of solar reforming.

## 3 Conclusion

We have reported floating carbon nitride photocatalysts for up‐scaled solar reforming of model and waste substrates using both Pt and a noble‐metal‐free co‐catalyst. HGM/CN*_x_* |Pt and HGM/CN*_x_* |Ni_2_P composites, where the role of the HGMs is as an inert low‐density support conferring floating in water, were prepared by a simple pyrolysis procedure followed by thermal or chemical reduction to deposit the co‐catalyst. Such floating composites and devices for solar fuel production enable versatile deployment scenarios, such as in turbid waste streams or on open waters, in addition to their advantages in terms of recyclability and scalability.^[^ ^34^ ^]^ Composite preparation and application conditions were optimized for H_2_ production and shown to generate H_2_ gas when coupled with ethylene glycol, pre‐treated PET, and pre‐treated cellulose. The floating composite was evaluated at small (5 mL, 4.9 cm^2^) and large (217 mL, 217 cm^2^) scales under vertical solar irradiation to simulate realistic application conditions, and it was found that the activity could be maintained over up to ten cycles (2 h per cycle). Estimated continuous catalyst reuse for scaled practical solar reforming is >1 year,^[^ ^10^ ^]^ necessitating long‐term evaluation of this composite in continuously operating systems before floating catalysts can be considered a solution to reuse, though the development of this floating platform is an important first step toward reaching such a goal. It was found that the noble‐metal co‐catalyst was substantially more robust than Ni_2_P under the application conditions tested and that only a very small Pt loading was required to achieve H_2_ production rates comparable to conventional CN*_x_* |Pt (0.033 ± 0.0013% m/m Pt in HGM/CN*_x_* |Pt vs 0.91 ± 0.017% m/m Pt in CN*_x_* |Pt). Recent work has estimated the costs of slurry catalyst materials TiO_2_|Pt (1% m/m Pt; $640 USD kg^−1^) and CN*_x_* |Ni_2_P (2% m/m Ni_2_P; $224 USD kg^−1^),^[^ ^27^ ^]^ and using the same cost basis, a CN*_x_* |Pt composite containing 0.04% m/m Pt would cost $224 USD kg^−1^. This brings the cost of a precious metal‐containing composite in line with the cost of a Ni_2_P co‐catalyst with higher solar reforming performance and greatly improves the cost compared to a CN*_x_* |Pt composite (1% m/m Pt; $800 USD kg^−1^). Another significant cost of solar reforming is based on the pre‐treatment process; the cost of NaOH for alkaline hydrolysis outweighs all other factors,^[^ ^10^ ^]^ therefore alternative pre‐treatment methods must be developed (e.g., neutral hydrothermal or saline hydrolysis)^[^ ^42^ ^]^ or solar reforming should initially be applied to waste streams that do not require pre‐treatment.^[^ ^19^ ^]^ Overall, the findings point toward the CN*_x_* composites in this work as a promising, scalable material for practical solar reforming that address the scaling challenges of reuse and light absorption by the waste solution by using small quantities of noble metals as co‐catalysts on a reusable floating platform.

## 4 Experimental Section

### Reagents

Cellulase (*Trichoderma reesei* ATCC 26 921), cellulose (20 µm, microcrystalline powder, 98%), chloroplatinic acid solution (8 wt% H_2_PtCl_6_ in H_2_O), ethylene glycol (99%), hydrochloric acid (37%), melamine (99%), nickel(II) chloride (98%), sodium acetate (≥99%), and sodium borohydride (99%) were purchased from Sigma‐Aldrich. PET (powder, 300 µm, >40% crystallinity, ≥99%) was purchased from Goodfellow Cambridge Ltd. Potassium hydroxide and sodium hypophosphite were purchased from Fisher Scientific. HGMs with an average diameter of 18 µm (3 M iM30k) were donated by Lawrence Industries. MilliQ water was used in all syntheses while deionized water was used in all substrate solutions and for washing procedures. TW solids were sourced from the middle fraction of a wet waste gravity separation process^[^ ^43^ ^]^ containing mixed plastics and refuse‐derived fuels and gifted by Montanuniversität Leoben, Austria. All chemicals were used as received.

### Preparation of Carbon Nitride and Floating Carbon Nitride Composite

CN*_x_* was prepared by heating melamine in a lidded crucible under air at 550 °C,^[^ ^44^ ^]^ and this procedure was modified for preparation of the floating CN*_x_* composite: HGMs (10 g) were thoroughly mixed with melamine at a specified mass ratio (1:1, 1:2, 1:3, 1:4; HGM:melamine), then heated at 1 °C min^−1^ to a final temperature of 550 °C for 3 h in an alumina crucible with a lid. After cooling, the resulting yellow solid (HGM/CN*_x_*) was collected and crushed to a fine powder using a mortar and pestle, then transferred to a 2 L separatory funnel. The HGM/CN*_x_* was washed repeatedly by gravimetric separation with deionized water to recover the floating fraction of the composite (by draining away any non‐floating solid with the aqueous phase) until a transparent, colorless aqueous phase was achieved with a floating layer of catalyst. This floating fraction was then drained from the funnel and dried at 80 °C overnight.

### Nickel Phosphide Deposition

HGM/CN*_x_* |Ni_2_P was produced using a Ni_2_P thermal reduction according to previous reports.^[^ ^9^, ^45^ ^]^ HGM/CN*_x_* was stirred in aqueous NiCl_2_∙6H_2_O and NaH_2_PO_2_ (15:5:1 HGM/CN*_x_*: NiCl_2_∙6H_2_O: NaH_2_PO_2_), dried under vacuum at 60 °C, annealed at 200 °C for 1 h under Ar, then washed with H_2_O by gravimetric separation (3×) and dried under vacuum at 60 °C.

### Platinum Deposition

CN*_x_* |Pt and HGM/CN*_x_* |Pt were produced using a literature‐adapted chemical reduction method.^[^ ^46^, ^47^ ^]^ 4 g of HGM/CN*_x_* (1.8 g CN*_x_*) was stirred in 120 mL H_2_O with 3.475 g trisodium citrate dihydrate and 420 µL H_2_[PtCl_6_] solution (8 wt%) for 30 min. Next, 60 mg of NaBH_4_ dissolved in 12 mL H_2_O was added dropwise and the suspension was stirred for an additional 30 min, then washed with H_2_O by gravimetric separation (3×) and dried at 80 °C overnight.

### Materials Characterization

Composite morphology and elemental analysis were assessed using a field‐emission scanning electron microscope (TESCAN MIRA3 FEG‐SEM) equipped with an Oxford Instruments Axtec Energy X‐maxN 80 EDX analysis system. SEM imaging and EDX were performed at 5 and 15 kV acceleration voltage, respectively. Morphology and composition were also assessed using STEM‐HAADF (FEI Talos F200X G2) and EDX using samples dried from aqueous suspension on a 300‐mesh lacey Cu grid. Inductively coupled plasma optical emission spectroscopy (ICP‐OES) was performed by the Microanalysis Service (Yusuf Hamied Department of Chemistry, University of Cambridge) using a Thermo Scientific iCAP 7400 ICP‐OES DUO. Samples containing Ni_2_P were digested overnight in 20% HNO_3_ (5 mg sample in 10 mL acid), then diluted 10× in H_2_O before analysis. Samples containing Pt were digested for 2 h at 80 °C in aqua regia (1:3 HNO_3_:HCl; 5 mg sample in 5 mL acid), then diluted 20× in H_2_O before analysis. CHN elemental analysis was also performed by the Microanalysis Service using an Exeter Analytical, Inc. CE‐440 Elemental Analyzer. UV/vis diffuse reflectance spectroscopy was performed using a Cary 60 UV/vis spectrophotometer with a Harrick Scientific Video Barrelino diffuse reflectance attachment.

### Substrate Pre‐Treatment

PET powder was pre‐treated according to a previously reported method using micronized powder or a plastic bottle (500 mL 30% RPET Juice Bottle, Ampulla Ltd.).^[^ ^16^ ^]^ PET powder (50 mg mL^−1^) was stirred in 2 mol L^−1^ KOH solution at 40 °C for 24 h, then filtered and the filtrate diluted to twice its volume with H_2_O, producing a hydrolyzed PET solution containing ethylene glycol (25 mg mL^−1^ PET_initial_) with a final KOH concentration of 1 mol L^−1^. The PET bottle (50 mg mL^−1^) was stirred in 2 mol L^−1^ KOH solution at 80 °C for 24 h, then filtered. Samples of the pre‐treated PET after filtration but before dilution (i.e., 50 mg mL^−1^ initial PET concentration) were gathered and diluted 10× in H_2_O (10% D_2_O) to analyze hydrolysate concentrations by water suppression NMR (Bruker 400 MHz Neo Prodigy). Both pre‐treated PET solutions contain ethylene glycol and terephthalic acid (TA) at concentrations of [EG]_micro_ = 8.6 mg mL^−1^, [TA]_micro_ = 23.7 mg mL^−1^, [EG]_bottle_ = 8.9 mg mL^−1^, and [TA]_bottle_ = 23.7 mg mL^−1^.

Cellulose was pre‐treated according to a previously reported method.^[^ ^48^ ^]^ Cellulose (40 mg mL^−1^) and cellulase (2 mg mL^−1^) were added to a 50 mmol L^−1^ sodium acetate solution titrated to pH = 5 with HCl and stirred at 37 °C. After 24 h, the mixture was heated to 90 °C and stirred for 15 min to denature the enzyme and then filtered. The filtrate was collected and analyzed by high‐performance liquid chromatography (Waters Breeze QS HPLC, Symmetry C18 300 Å 4.6×75 mm reverse phase column) against standards of glucose and cellobiose to determine concentrations (10.6 mg mL^−1^ glucose; 6.9 mg mL^−1^ cellobiose; Figure S1, Supporting Information) and refrigerated until used.

TW suspensions were prepared by stirring mixed waste (25 g; recovered from the middle fraction of a floatation separation process) intended as combustible refuse‐derived fuel in 1 L of 1 mol L^−1^ KOH at 80 °C for 24 h, followed by coarse filtration through glass wool to remove large particles. TW+EG was prepared by adding EG to this TW suspension at a concentration of 25 mg mL^−1^ and TW+PET was prepared by adding pretreated PET (plastic bottle) to an estimated PET concentration of 37.5 g L^−1^ (≈6.9 mg mL^−1^ EG, ≈19.2 mg mL^−1^ TA).

### Small Vial Solar Reforming Experiments

The catalyst (3 mg CN*_x_*) was added to 2 mL of 1 mol L^−1^ KOH solution containing 25 mg mL^−1^ ethylene glycol and 1.6 µL H_2_PtCl_6_ solution (8 wt%) for in situ photodeposition of metallic Pt upon light exposure in a 7.9 mL pyrex vial. In the case of HGM/CN*_x_* composites, an amount was added such that the final mass of CN*_x_* in the experiment was 3 mg. In the case of turbid waste experiments, CN*_x_* |Pt and HGM/CN*_x_* |Pt already loaded with metallic Pt by chemical reduction were used, and thus no H_2_PtCl_6_ solution was added to the SR solution. A stir bar was added and the vial was sealed with a septum. This suspension was purged with N_2_/CH_4_ gas (98/2% v/v) for 10 min, then irradiated by a solar light simulator (Newport Oriel, 100 mW cm^−2^) with an air mass 1.5 global (AM1.5G) filter and a water filter to remove infrared radiation. All samples were irradiated for 2 h while stirring at 600 rpm and maintained at 25 °C using a water bath and chiller.

### Small Reactor Solar Reforming Experiments (4.9 cm^2^)

The catalyst (7.5–240 mg HGM/CN*_x_* |Ni_2_P or HGM/CN*_x_* |Pt) was added to 5 mL of 1 mol L^−1^ KOH solution containing 25 mg mL^−1^ EG or pre‐treated PET, or 5 mL of 50 mmol L^−1^ pH 5 sodium acetate solution containing 25 mg mL^−1^ pre‐treated cellulose in a small glass reactor (V = 50 mL, SA = 4.9 cm^2^; Figure S2, Supporting Information). The vessels were sealed with septa, then the suspension was purged with N_2_/CH_4_ gas (98/2% v/v) for 10 min. The reactors were vertically irradiated by a solar light simulator (G2V SunBrick, 100 mW cm^−2^) with LED output calibrated to AM1.5G for 2 h. Samples were not stirred or temperature controlled. For consecutive solar reforming trials, the reactors were opened immediately following gas sampling, the aqueous solution was aspirated from the bottom of the reactor using a needle (19G) and syringe, then fresh substrate solution (5 mL 1 mol L^−1^ KOH, 25 mg mL^−1^ EG or PET; or 50 mmol L^−1^ sodium acetate, 25 mg mL^−1^ cellulose) was added to the reactor which was subsequently sealed, purged with N_2_/CH_4_, and irradiated with solar light as described above. Each recyclability experiment consisted of ten consecutive runs performed in triplicate.

### Large Reactor Solar Reforming Experiments (217 cm^2^)

The catalyst (2.609 g HGM/CN*_x_* |Ni_2_P or HGM/CN*_x_* |Pt) was added to 217 mL of 1 mol L^−1^ KOH solution containing 25 mg mL^−1^ EG or pre‐treated PET in a large reactor (V = 1.5 L; Figure S2, Supporting Information) constructed using PVC, which has good stability in alkaline conditions.^[^ ^49^ ^]^ The vessel was sealed with a septum (inserted into a hole on the side of the reactor facilitating gas extraction and purging processes), then the suspension was purged with N_2_/CH_4_ gas (98/2% v/v) for 45 min. The reactor was vertically irradiated for 2 h periods by a solar light simulator (G2V SunBrick, 100 mW cm^−2^) with the LED output calibrated to AM1.5G. Samples were not stirred or temperature controlled. For consecutive solar reforming trials, the reactor was opened immediately following gas sampling, the aqueous solution was aspirated from the bottom of the reactor using a needle (19G) and syringe, leaving behind the floating composite, then fresh substrate solution (217 mL 1 mol L^−1^ KOH, 25 mg mL^−1^ EG or PET) was added to the reactor which was subsequently sealed, purged with N_2_/CH_4_, and irradiated with solar light as described above. Each recyclability experiment consisted of ten consecutive runs performed in triplicate.

### Gas Chromatography

The H_2_ concentration from SR experiments was quantified from the headspace gas after 2 h irradiation by manual injection into a Shimadzu GC‐2010 Plus gas chromatograph (GC; Dielectric‐barrier discharge Ionization Detector (BID), He carrier gas) and quantified using methane as an internal standard (2% of headspace). Evolution of methane was evaluated in representative reactions (1.5 mg mL^−1^ CN*_x_* |Pt, 1 mol L^−1^ KOH, 25 mg mL^−1^ EG, 1 h; 1.5 mg mL^−1^ CN*_x_* |Pt, 50 mmol L^−1^ Na acetate, pre‐treated cellulose, 1 h) compared to an external standard (2% CH_4_ in N_2_) and found to be negligible (59 nmol L^−1^ and 2.7 µmol L^−1^, respectively) compared to the concentration of the internal standard (816 µmol L^−1^) used in other tests.

### Treatment of Data

All gas chromatography measurements were performed in triplicate unless otherwise stated, and presented as the unweighted mean of the three measurements ± standard deviation (*σ*). SR Activity was calculated from the molar concentration of H_2_ present in the headspace gas normalized by time and reactor area or catalyst mass and reported as “areal activity” (µmol_H2_ m^−2^ h^−1^) or “specific activity” (µmol_H2_ g_CN_ *_x_* ^−1^ h^−1^). Percent activity (%), used for comparisons within recyclability studies, is given by *A*_i_ */A*_0_ where *A*_i_ is the SR activity of run *i* and *A*_0_ is the activity of the first run. *σ* was calculated as $\sigma =\sqrt{\frac{\sum {\left(x-\bar{x}\right)}^{2}}{n\;-\;1}}$ where *n* is the number of replicates, *x* is the value of a single measurement, and $\bar{x}$ is the unweighted mean of the measurements. The standard deviation for bar charts is presented as a black error bar with flat caps and presented as the calculated standard deviation or 5% of the value of the bar, whichever is larger, while for line graphs it is represented as a shaded area surrounding the line and data points.

## Conflict of Interest

The authors declare no conflict of interest.

## Supporting information

Supporting Information

## Data Availability

The data that support the findings of this study are openly available in the Cambridge Data repository at http://doi.org/10.17863/CAM.96335
