# Supplementary material for: Floating Carbon Nitride Composites for Practical Solar Reforming of Pre‐Treated Wastes to Hydrogen Gas
Source: Adv Sci (Weinh). 2023 May 12;10(21):2207314. doi: 10.1002/advs.202207314 (PMC10375181; doi:10.1002/advs.202207314)
Supplement: Supplementary file 1 — Supporting Information [file ADVS-10-2207314-s001.pdf]

ADVANCED SCIENCE
Open Access

Supporting Information

for *Adv. Sci.*, DOI 10.1002/advs.202207314

Floating Carbon Nitride Composites for Practical Solar Reforming of Pre-Treated Wastes to Hydrogen Gas

*Stuart Linley and Erwin Reisner**

Supporting Information for

Floating Carbon Nitride Composites for Practical Solar Reforming of Solid Wastes to Hydrogen Gas

Stuart Linley[1] and Erwin Reisner[1,*]

[1]Yusuf Hamied Department of Chemistry, University of Cambridge, Cambridge, UK, Website: http://www-reisner.ch.cam.ac.uk; [*]Corresponding author Email: reisner@ch.cam.ac.uk

Alternative composite compositions

Experimental Section

Nitrogen-doped carbon dots (N-g-CDs) were prepared from a procedure by Achilleos et al.[1] by heating 10 g of aspartic acid in a 250 mL pyrex beaker at 320 °C for 100 h (ramp rate of 10 °C/min). After cooling to room temperature, 200 mL of Millipore DI water was added to the beaker, followed by 5 mL of 4 mol L^{-1} NaOH and stirred overnight. The following day, the pH of the N-g-CD suspension was reduced to 6.9 using 0.6 mL 2 mol L^{-1} H_2SO_4, diluted by 3.33 times, and freeze dried over 3 days before further use.

The aluminosilicate cement was prepared using a procedure adapted from Brace and Matijevic[2]. First, 1.140 g sodium metasilicate (Na_2SiO_3) and 1.875 g aluminum nitrate nonahydrate ($Al(NO_3)_3 \cdot 9H_2O$) were weighed out in separate 50 mL beakers and dissolved in 15 mL or 5 mL H_2O, respectively. After dissolved, the aluminum nitrate solution was poured into the sodium silicate solution while stirring (~500 rpm), precipitating a white gel (aluminum/silicon hydroxides). 7 mL sulfuric acid (H_2SO_4; 2 mol L^{-1}) was slowly added until the resulting mixture appeared translucent. The pH of this solution was then adjusted to 3.5 by adding 3 mL of 4 mol L^{-1} sodium hydroxide (NaOH) and left to stir. 40 mL of floating support material (10 g of iM30k) and an amount of photocatalyst (P25 TiO_2 (Aeroxide®, Evonik GmbH) or N-g-CDs) were then added to the pH 3.5 aluminosilicate mixture and stirred for 30 minutes, after which the resulting suspension was poured into a petri dish and dried at 100 °C overnight. After drying, the cake was recovered, weighed, powderised, and washed 3x with H_2O by floatation.

Hollow glass microsphere (HGM) durability

After the preparation process described in the experimental section, the aluminosilicate-coated HGM samples were weighed (before and after washing) to determine the proportion of the material that remained floating after coating and powderisation (Table S9). It was found that the lower density glass bubbles did not retain much floatation after processing, and only the denser, smaller iM30k and iM16k glass bubbles demonstrated sufficient durability to warrant application in a recyclable floating composite.

TiO_2 and carbon dots were also incorporated into the aluminosilicate cement and examined as a photoreforming catalyst using Pt as a co-catalyst. In the case of TiO_2, the recyclability of the composite was not promising as the activity in vertically illuminated small reactor systems rapidly declined over 10 consecutive cycles, reaching a minimum of 21.7% (12 mg mL^{-1} composite) and 5.7% (48 mg mL^{-1} composite) of the initial activity depending on the loading of the composite (Figure S22). The carbon dot-based composite did not demonstrate any photoreforming activity when using deposited Pt or Ni_2P co-catalysts, and a functioning, recyclable photoreforming composite based on carbon dots was not successfully produced. Hydrogen evolution activity of floating carbon dot composites without a deposited co-catalyst was achieved using a DuBois-type molecular catalyst (**NiP**) and a sacrificial electron donor (Ethylenediaminetetraacetic acid)[3], though this was not considered to be of interest for a scalable, recyclable photoreforming system.

Supporting Tables

Table S1. Floatation of various perlite and hollow glass microsphere samples after three consecutive washes with DI water. The sample with the highest %Float after three washes was determined to be the most durable.

Support Type	Sample	Initial Mass (g)	Mass after wash (g)	%Float	%Sink
**Perlite**	P3LW	8.05	4.65	57.7	42.3
	P05	28.90	2.92	10.1	89.9
**Hollow Glass Microspheres**	iM16k	108.78	103.95	95.6	4.4
	iM30k	134.06	127.67	96.7	3.3
	K1	30.80	23.01	74.7	25.3
	K15	35.86	29.01	82.8	17.2
	S38HS	99.98	85.00	85.0	15.0

Table S2. HGM/CN_x composite solar reforming activity in small vials (2 mL, 1 mol L^{-1} KOH, 25 mg mL^{-1} ethylene glycol, *in situ* photodeposition of Pt from 1.6 µL H_2PtCl_6 8% wt. solution, 1.5 mg_{CNx} mL^{-1}, 100 mW cm^{-2} side illumination, stirred, 2 h) dependence on synthesis conditions.

HGM:Melamine Ratio	Specific Activity (μmol_{H2} g_{CNx}^{-1} h^{-1})
**1:1**	71.1 ± 9.2
**1:2**	95.3 ± 3.4
**1:3**	124.0 ± 16.1
**1:4**	137.0 ± 11.4
**CN_x (control)**	95.1 ± 9.1

Table S3. Small vial control experiments (2 mL, 1.5 mg_{CNx} mL^{-1}, 100 mW cm^{-2} side illumination, stirred, 2 h). Samples 1-6 used 25 mg mL^{-1} ethylene glycol (EG) in 1 mol L^{-1} KOH. Samples 7-8 were performed in 50 mmol L^{-1} Na-acetate at pH = 5, sample 8 containing pre-treated cellulose (cellulose hydrolysates: 10.6 mg mL^{-1} glucose, 6.9 mg mL^{-1} cellobiose). Samples 9-10 used pre-treated PET in 1 mol L^{-1} KOH (PET hydrolystates: ~4.3 mg mL^{-1} EG, ~11.9 mg mL^{-1} terephthalic acid). Pt content was measured by ICP-OES after each SR experiment. *n.d.* denotes 'not detected' and *n.m.* denotes 'not measured'.

Sample	Composite	Cocatalyst	Substrate	Specific Activity (μmol_{H2} g_{CNx}^{-1} h^{-1})	Pt content (% wt.)
**1**	CN_x	Pt	EG	166.5 ± 30.1	0.91 ± 0.017
**2**	CN_x	H_2PtCl_6	EG	95.1 ± 9.1	0.19 ± 0.058
**3**	HGM/CN_x	Pt	EG	127.5 ± 26.9	0.033 ± 0.0013
**4**	HGM/CN_x	H_2PtCl_6	EG	124.0 ± 16.1	0.13 ± 0.018
**5**	HGM/CN_x	None	EG	*n.d.*	*n.m.*
**6**	HGM/CN_x	Pt	None	0.41 ± 0.36	*n.m.*
**7**	CN_x	Pt	Na-acetate	2.5 ± 1.8	*n.m.*
**8**	CN_x	Pt	Cellulose	29.2 ± 1.9	*n.m.*
**9**	HGM/CN_x*	Pt	PET	78.2 ± 7.5	*n.m.*
**10**	HGM/CN_x†	Pt	PET	99.6 ± 2.6	*n.m.*

*****HGM/CN_x sample before application in SR experiment
†HGM/CN_x sample after 10 cycles SR in large reactor

Table S4. HGM/CN_x composite solar reforming activity in small reactors under different composite loading concentrations (5 mL, 1 mol L^{-1} KOH, 25 mg mL^{-1} ethylene glycol, 100 mW cm^{-2} vertical illumination, no stirring, 2 h). Error is given as standard deviation over a triplicate.

Composite	Composite Concentration (mg mL^{-1})	Specific Activity (μmol_{H2} g_{CNx}^{-1} h^{-1})	Areal Activity (μmol_{H2} m^{-2} h^{-1})
HGM/CN_x|Pt	**1.5**	16.7 ± 3.7	119.2 ± 25.6
	**3**	16.5 ± 2.1	225.4 ± 22.7
	**6**	19.8 ± 5.8	554.9 ± 140.0
	**12**	15.6 ± 3.8	855.2 ± 189.1
	**24**	10.0 ± 1.2	1109.0 ± 115.5
	**48**	4.9 ± 2.2	921.3 ± 158.9
HGM/CN_x|Ni_2P	**1.5**	9.6 ± 1.0	67.8 ± 1.5
	**3**	10.0 ± 2.7	139.8 ± 39.3
	**6**	8.4 ± 1.6	228.8 ± 44.0
	**12**	6.3 ± 2.2	349.0 ± 120.7
	**24**	4.5 ± 1.0	490.8 ± 113.3
	**48**	2.0 ± 0.3	445.1 ± 61.1

Table S5. HGM/CN_x composite recovery after 10 consecutive solar reforming trials

Sample	Initial Mass	Final Mass	Recovered
Small Reactors (SA = 4.9 cm^2)	(mg)	(mg)	(%)
HGM/CN_x|Ni_2P, EG	60.1 ± 0.3	39.0 ± 0.7	64.9 ± 1.2
HGM/CN_x|Ni_2P, PET	60.1 ± 0.2	29.0 ± 1.3	48.2 ± 2.1
HGM/CN_x|Ni_2P, Cellulose	60.1 ± 0.3	33.9 ± 1.6	56.3 ± 2.7
HGM/CN_x|Pt, EG	59.9 ± 0.2	39.2 ± 3.2	65.4 ± 5.4
HGM/CN_x|Pt, PET	59.8 ± 0.2	34.8 ± 2.1	58.2 ± 3.6
HGM/CN_x|Pt, Cellulose	59.9 ± 0.5	36.3 ± 0.6	60.6 ± 1.0
Large Reactor (SA = 217.4 cm^2)	(g)	(g)	(%)
HGM/CN_x|Ni_2P, EG	2.611 ± 0.004	1.668 ± 0.220	63.9 ± 8.4
HGM/CN_x|Ni_2P, PET	2.613 ± 0.005	1.772 ± 0.154	67.8 ± 5.9
HGM/CN_x|Pt, EG	2.609 ± 0.006	1.855 ± 0.119	71.1 ± 4.6
HGM/CN_x|Pt, PET	2.610 ± 0.001	1.871 ± 0.084	71.7 ± 3.2

Table S6. HGM/CN_x composite solar reforming activity in small reactors over 10 consecutive recycles (5 mL, 1 mol L^{-1} KOH, 25 mg mL^{-1} substrate, 100 mW cm^{-2} vertical illumination, no stirring, 2 h). Error is given as standard deviation over a triplicate; *n.d.* denotes 'not detected'.

**Substrate**	**Co-catalyst**	**Consecutive Recycle Areal Activity (μmol_{H2} m^{-2} h^{-1})**									
		1	2	3	4	5	6	7	8	9	10
**EG**	Pt	1411.9 ± 134.3	1556.0 ± 223.3	1571.2 ± 129.5	1585.9 ± 155.0	1445.6 ± 116.2	1384.7 ± 86.1	1107.6 ± 24.6	1251.6 ± 81.2	1238.4 ± 87.9	1162.7 ± 14.8
	Ni_2P	268.3 ± 44.0	266.5 ± 54.9	263.9 ± 82.9	190.9 ± 36.6	181.2 ± 58.9	168.9 ± 19.1	112.1 ± 32.5	142.9 ± 43.5	138.7 ± 57.1	83.4 ± 32.5
**PET**	Pt	846.7 ± 19.2	815.8 ± 44.0	843.6 ± 160.7	666.8 ± 216.8	622.6 ± 125.3	626.4 ± 59.3	634.4 ± 124.5	458.8 ± 128.2	380.0 ± 103.9	330.6 ± 104.0
	Ni_2P	54.5 ± 14.9	56.7 ± 13.6	53.2 ± 7.6	36.3 ± 5.5	21.5 ± 3.3	14.4 ± 1.4	14.0 ± 2.5	18.7 ± 4.9	8.7 ± 1.6	10.7 ± 0.6
**Cellulose**	Pt	144.2 ± 18.3	171.7 ± 29.3	163.6 ± 37.1	169.8 ± 32.3	163.3 ± 34.1	138.0 ± 27.6	129.0 ± 25.8	34.3 ± 10.5	111.9 ± 32.1	97.3 ± 16.5
	Ni_2P	72.8 ± 3.1	16.0 ± 2.5	*n.d.*	4.2 ± 0.7	*n.d.*	*n.d.*	*n.d.*	*n.d.*	*n.d.*	*n.d.*

Table S7. HGM/CN_x composite solar reforming activity in large reactor over 10 consecutive recycles (217 mL, 1 mol L^{-1} KOH, 25 mg mL^{-1} substrate, 100 mW cm^{-2} vertical illumination, no stirring, 2 h). Error is given as standard deviation over a triplicate.

**Substrate**	**Co-catalyst**	**Consecutive Recycle Areal Activity (μmol_{H2} m^{-2} h^{-1})**									
		1	2	3	4	5	6	7	8	9	10
**EG**	Pt	2190.9 ± 80.5	2557.8 ± 196.6	2241.3 ± 292.2	2213.9 ± 435.6	1943.7 ± 365.1	1916.6 ± 269.8	2073.0 ± 151.0	1867.7 ± 312.3	1915.6 ± 227.2	2072.8 ± 157.7
	Ni_2P	560.6 ± 182.3	640.3 ± 132.2	537.4 ± 95.9	380.2 ± 25.8	333.0 ± 91.4	233.5 ± 5.9	193.0 ± 16.5	142.7 ± 27.7	122.7 ± 12.0	94.8 ± 15.7
**PET**	Pt	1064.5 ± 101.5	133.8 ± 240.0	1262.8 ± 56.4	1304.6 ± 111.3	1266.4 ± 95.0	1208.1 ± 8.3	1158.8 ± 120.6	1150.3 ± 56.3	1099.5 ± 34.1	994.5 ± 121.1
	Ni_2P	369.4 ± 158.3	350.3 ± 248.8	290.6 ± 204.5	249.3 ± 155.4	205.5 ± 83.9	152.8 ± 47.5	125.3 ± 73.1	149.2 ± 67.7	97.5 ± 74.3	106.9 ± 53.3

Table S8. HGM/CN_x composite carbon nitride content of floating particles before and after use

Sample	Floating particles recovered (%)	Initial CN_x content (%)	Recovered particle CN_x content (%)
**iM30k/CN_x|Pt (large reactor, 10 cycles)**	71.1 ± 4.6	46.6 ± 0.1	44.6 ± 0.1
**iM30k/CN_x|Ni_2P (large reactor, 10 cycles)**	63.9 ± 8.4	44.0 ± 0.0	42.8 ± 0.1
**iM30k/CN_x 30 min stirring***	75.9 ± 2.6	45.7 ± 0.1	45.0 ± 0.1
**iM30k/CN_x 60 min stirring***	85.3 ± 4.7	45.7 ± 0.1	49.1 ± 0.1

***stirred at 500 rpm, 100 mg iM30k/CN_x in 20 mL H_2O, 2 cm stir bar in 50 mL beaker

Table S9. Aluminosilicate-coated HGM samples floatation and durability. The floating support candidates with the greatest durability and fraction of floating particles are ranked on a colour scale (red = least durable, green = most durable).

Glass Bubbles	m_{HGM}	$m_{Al:Si/HGM}$	$m_{Al:Si}$	$m_{floating}$	$m_{sinking}$	floating fraction	sinking fraction
**K1**	1.9549	5.3674	3.4125	0.4226	4.9448	7.9%	92.1%
**K15**	2.7738	5.4367	2.6629	0.7104	4.7263	13.1%	86.9%
**S38HS**	6.65	10.1935	3.5435	3.555	6.6385	34.9%	65.1%
**iM16k**	8.2728	11.8276	3.5548	7.9511	3.8765	67.2%	32.8%
**iM30k**	10.08	13.794	3.714	9.597	4.197	69.6%	30.4%

Supporting Figures

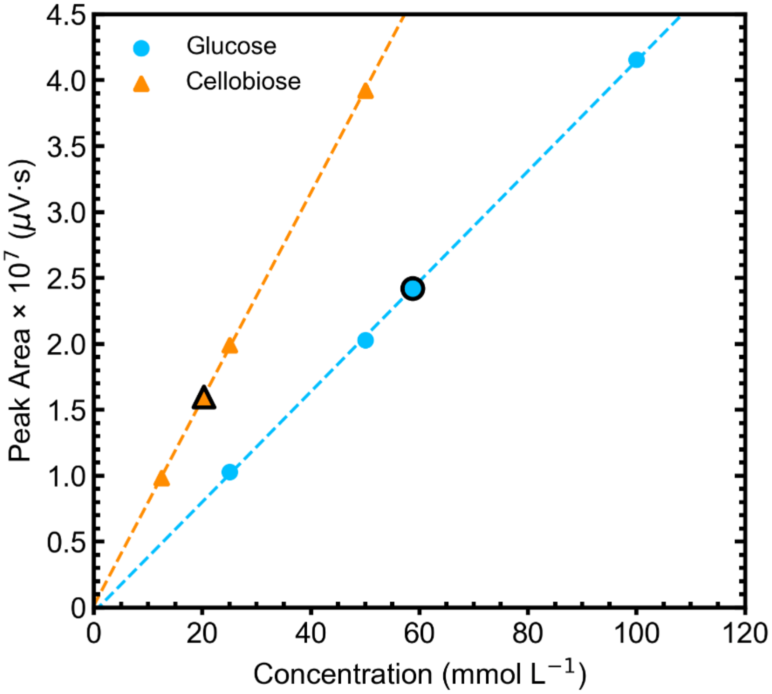

Figure S1. High performance liquid chromatography (HPLC) analysis of pre-treated cellulose (black outline) against glucose and cellobiose standards. Retention time for glucose and cellobiose in pretreated cellulose was 13.77 and 11.28 minutes, respectively, versus 13.90 ± 0.08 and 11.40 ± 0.10 minutes for each standard.

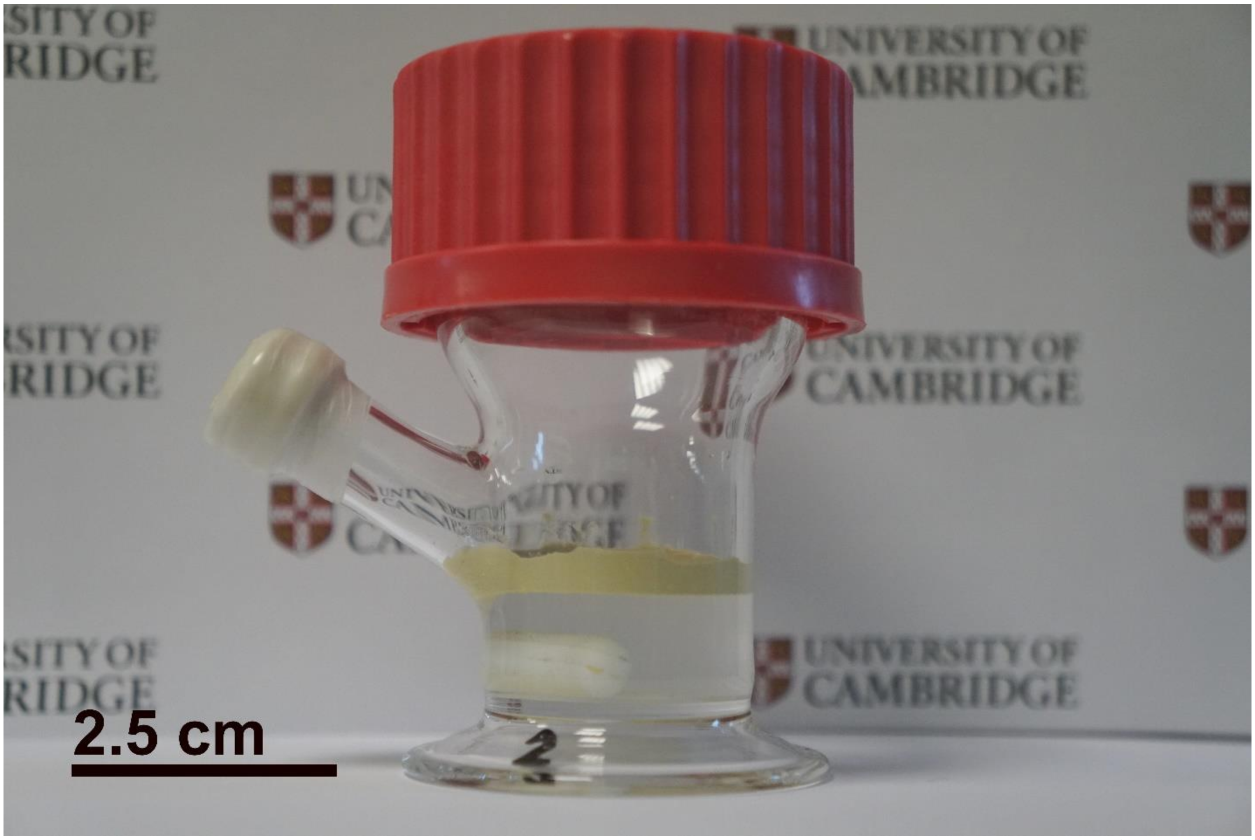

Figure S2. Side view of small reactor (base diameter 2.5 cm, V = 50 mL, SA = 4.9 cm^2) containing HGM/CN_x, water (5 mL), and a stir bar.

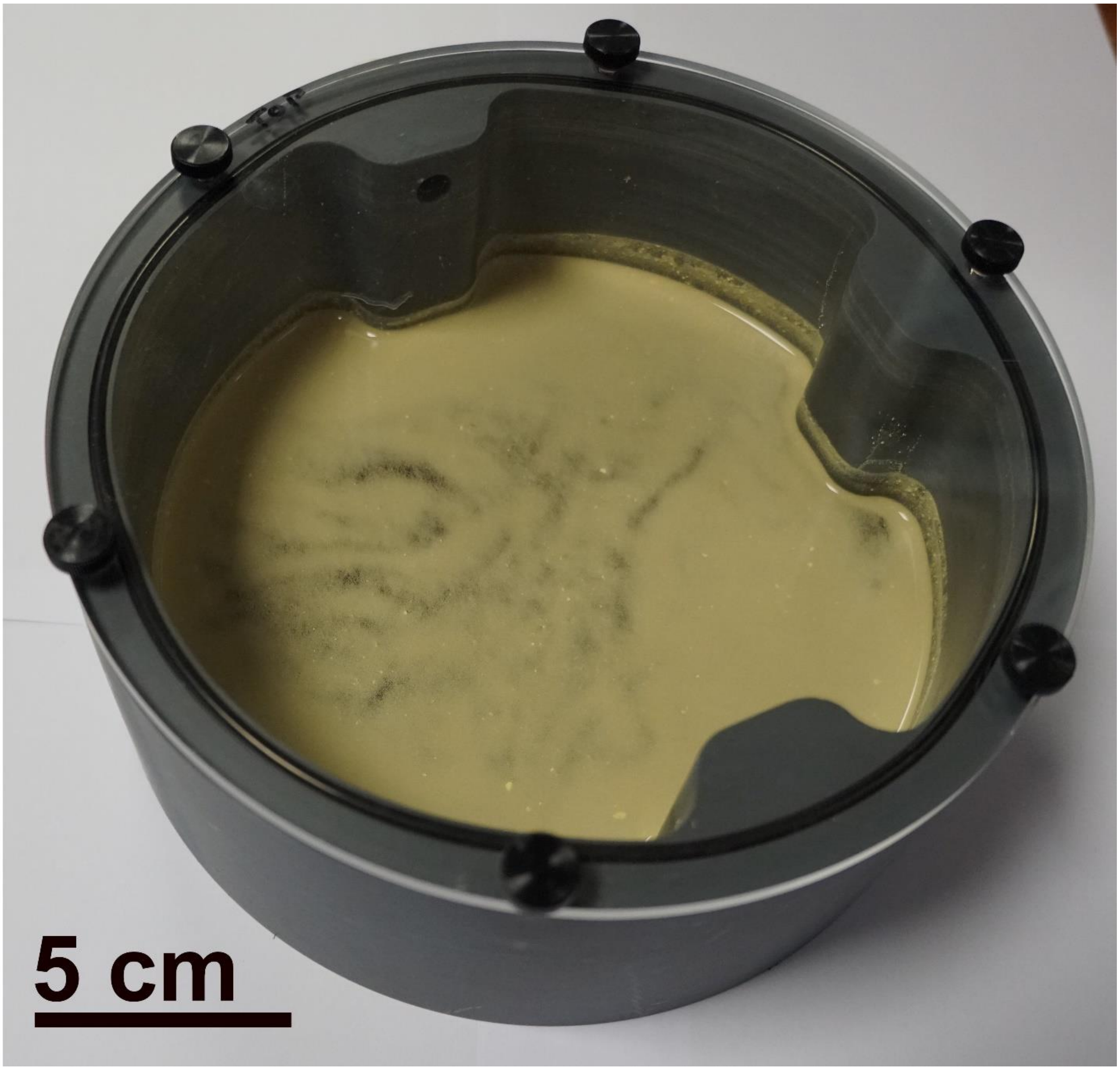

Figure S3. Oblique view of large reactor (inner diameter 18 cm, V = 1.5 L, SA = 217.4 cm^2) containing HGM/CN_x|Pt and turbid waste solution spiked with 25 mg mL^{-1} ethylene glycol (217 mL).

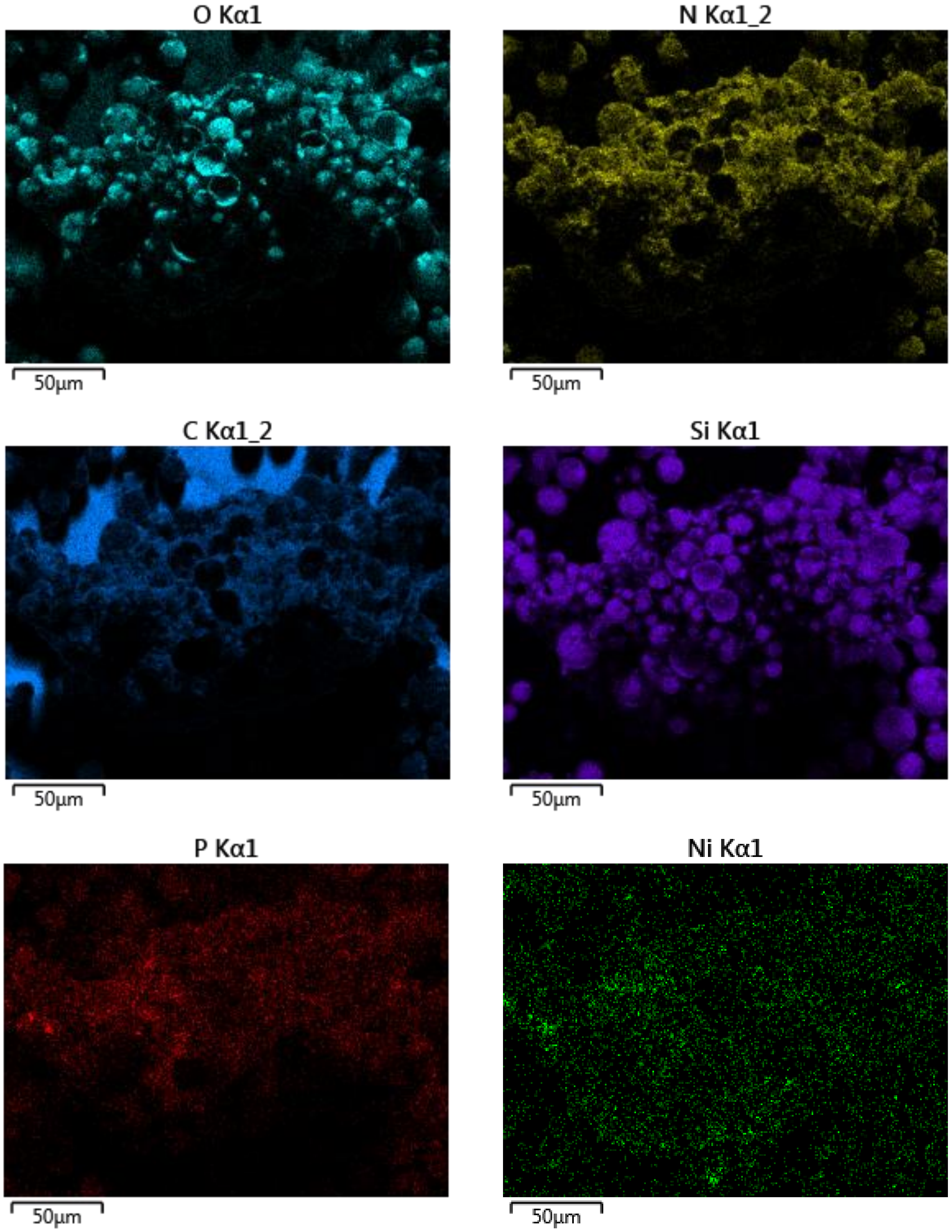

Figure S4. SEM-EDX element maps of HGM/CN_x|Ni_2P sample seen in Figure 2E. Elemental content (% wt.) was reported as O: 21.5%, N: 29.9%, C: 33.9%, Si: 9.7%, P: 0.3%, and Ni: 1.0%, with small amounts of Ca, Na, and S making up the remaining weight.

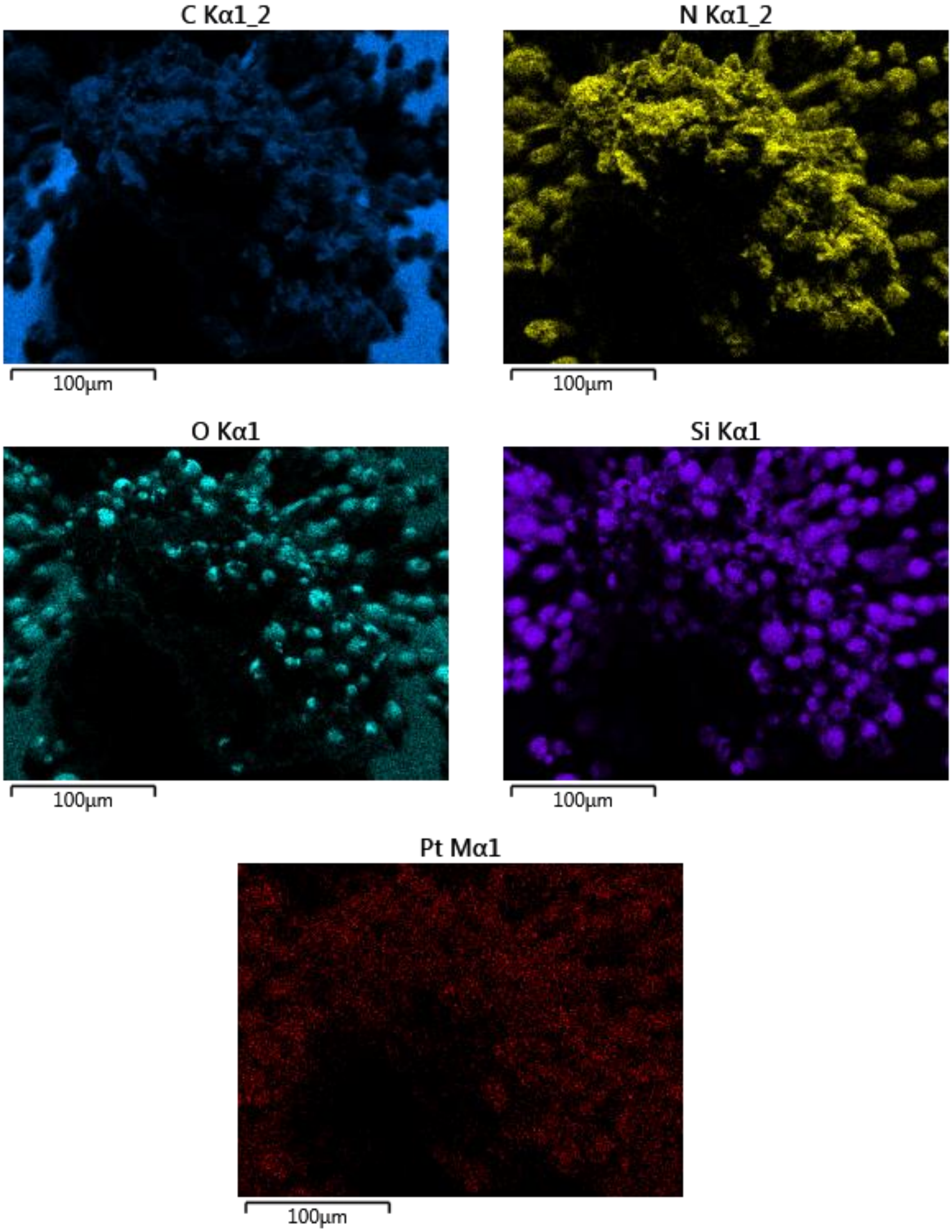

Figure S5. SEM-EDX element maps of HGM/CN_x|Pt sample seen in Figure 2F.

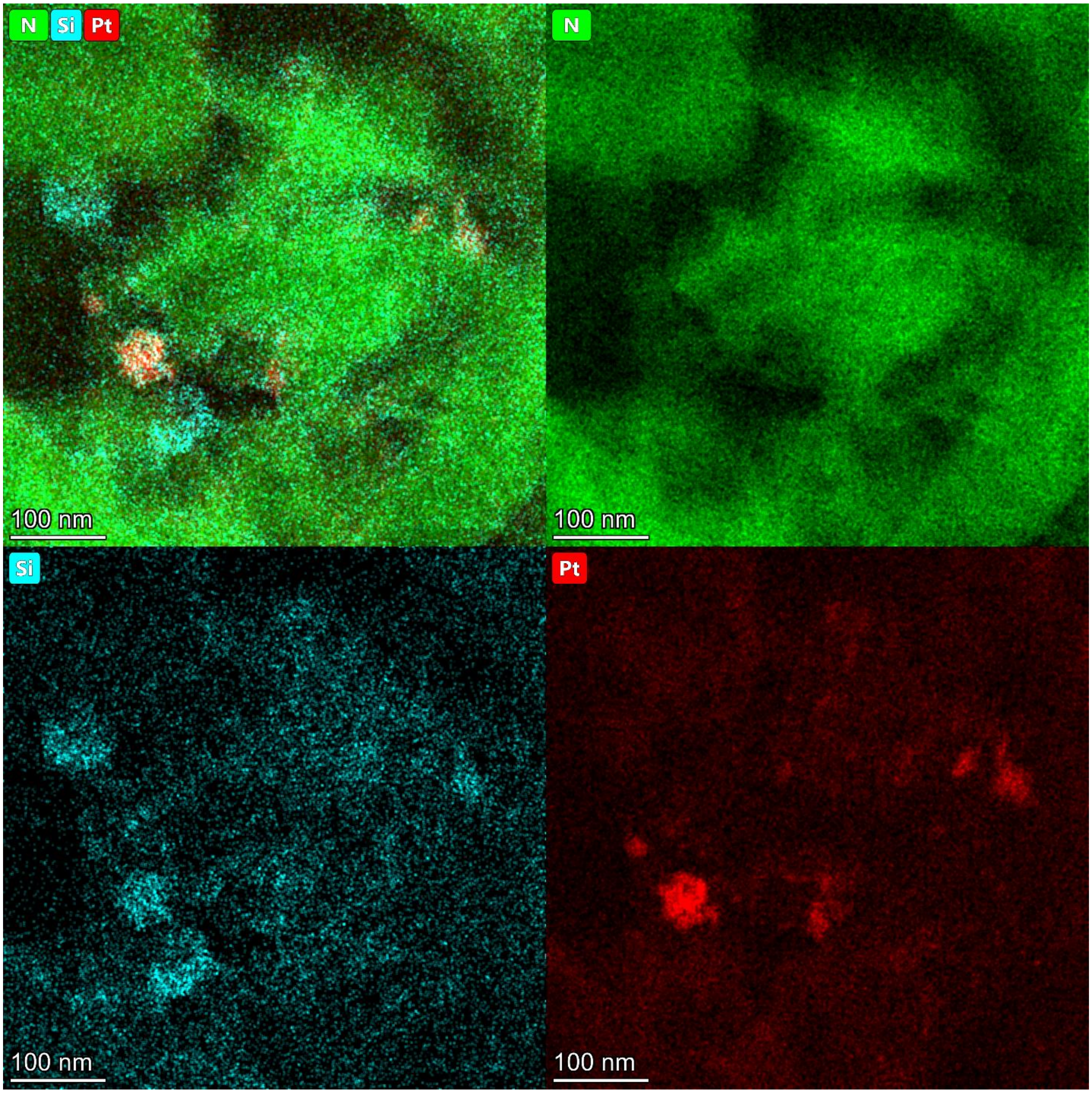

Figure S6. STEM/EDX maps of HGM/CN_x|Pt demonstrating the distribution of Pt across the composite surface.

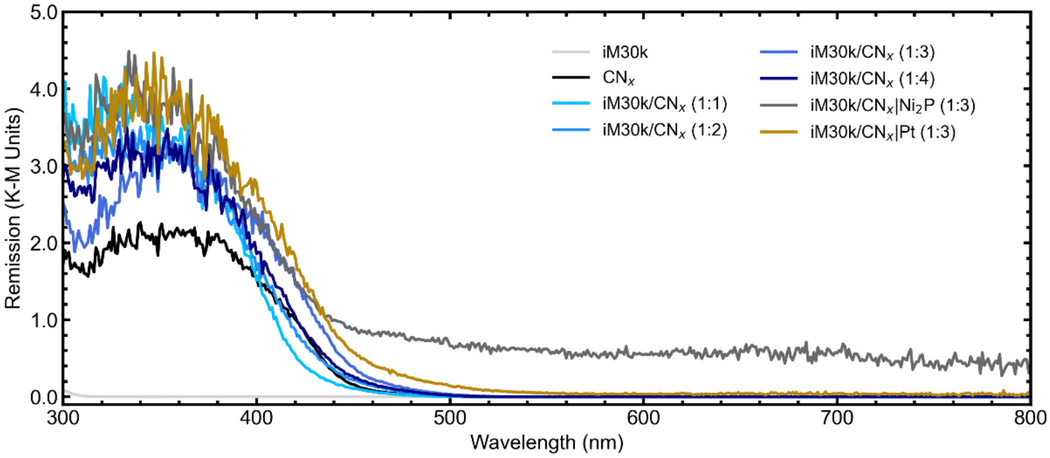

Figure S7. UV-vis remission spectra (Kubelka-Munk) of the floating carbon nitride composites and their components.

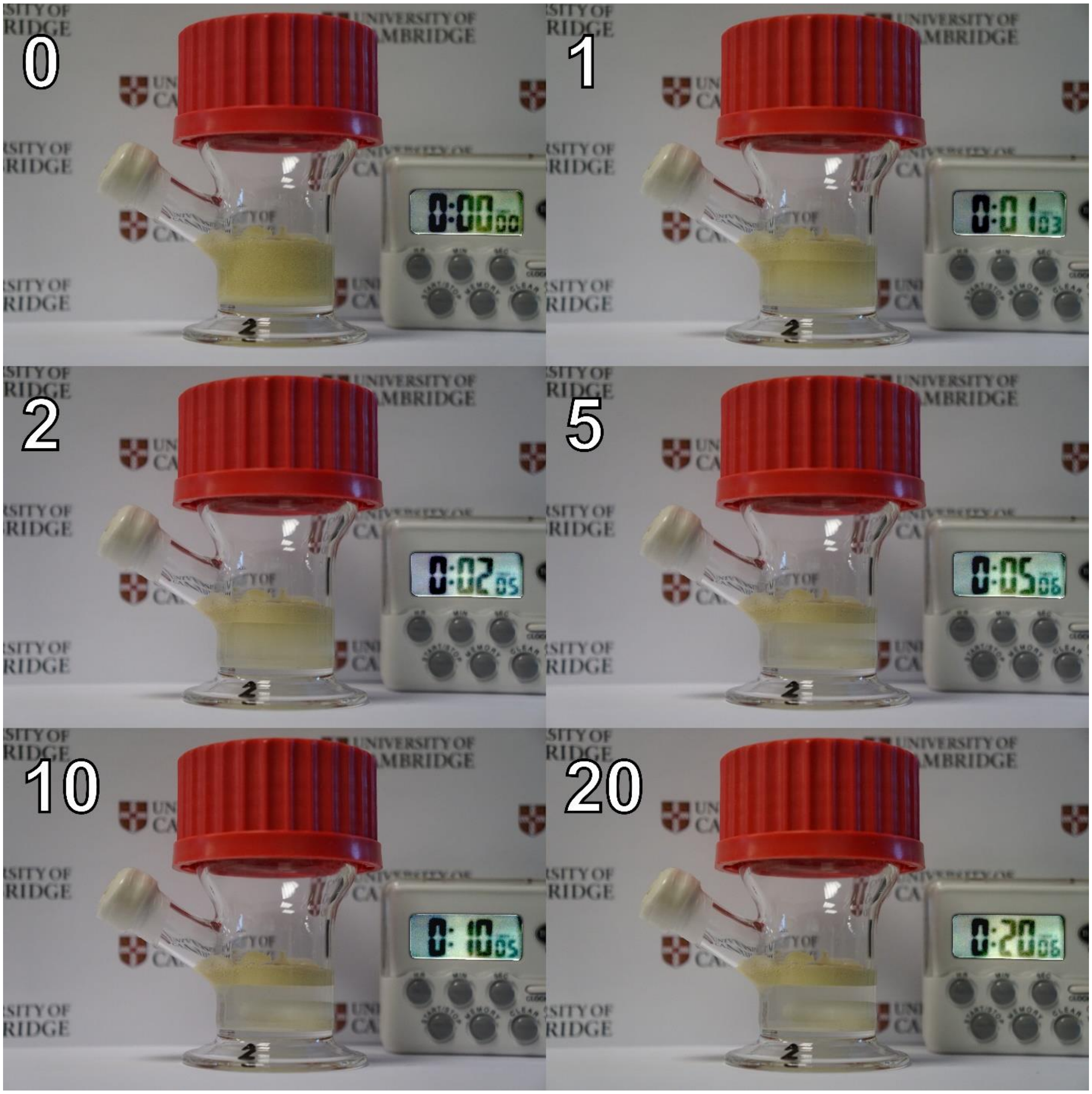

Figure S8. Separation of HGM/CN_x from 5 mL water in a small reactor. The number in the upper left of each panel represents the time in minutes after stirring at 300 rpm. A timer showing the time elapsed after stirring is visible in the bottom right of each panel. The brightness and contrast of the timer screens have been increased to allow the timestamps to be clearly seen.

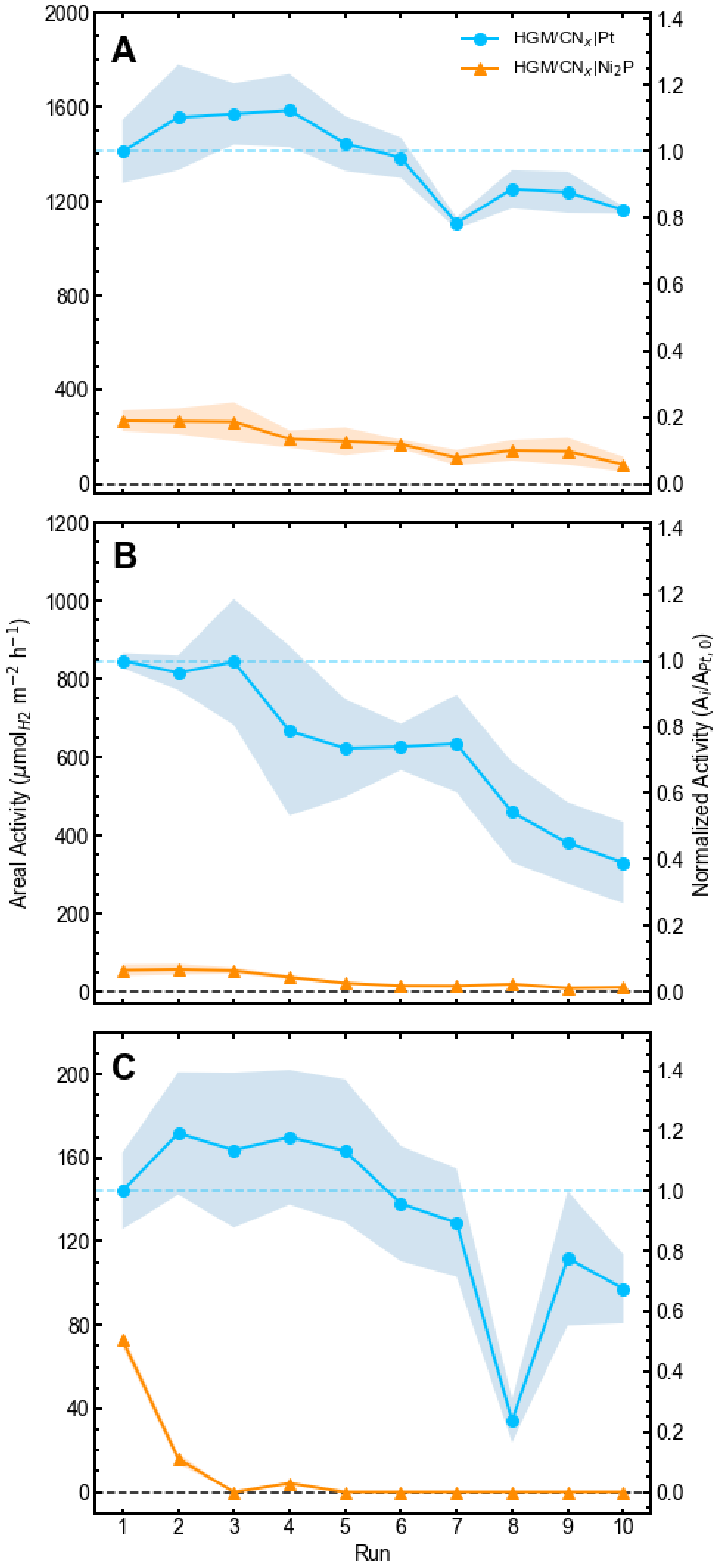

Figure S9. Figure 4 from main text including the outlying data points from run 7 in EG and run 8 in pre-treated cellulose with HGM/CN$_x$|Pt composites.

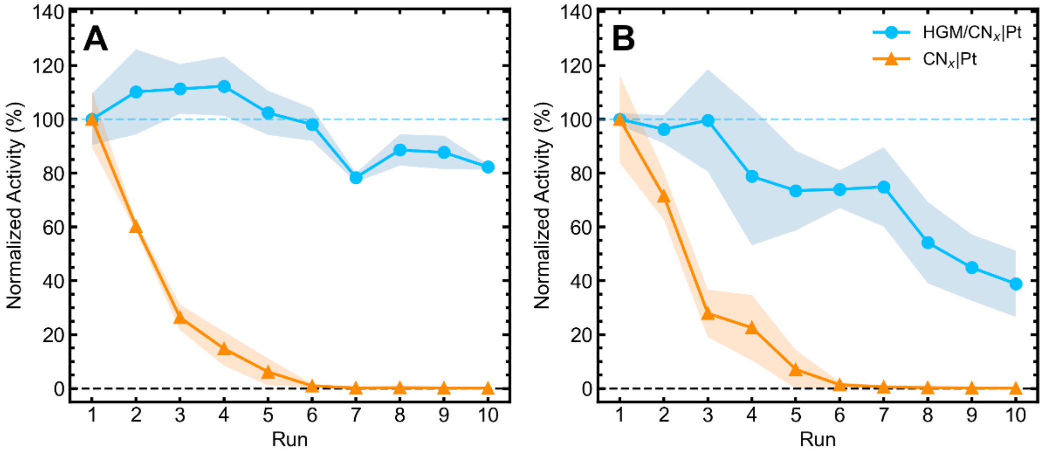

Figure S10. Small area (SA = 4.9 cm^2) solar reforming activity of floating (HGM/CN_x|Pt) and non-floating (CN_x|Pt) composites using top-down irradiation (100 mW cm^{-2}, V = 5 mL, 1 mol L^{-1} KOH, [substrate] = 25 mg mL^{-1}, no stirring, 2h) over 10 consecutive trials with different substrates: **A** Ethylene glycol, **B** Polyethylene terephthalate). The shaded area surrounding each line shows the standard deviation from each triplicate. The black dashed line at the bottom of the graph shows zero and the blue dashed line shows initial activity of 100% (A/A_0).

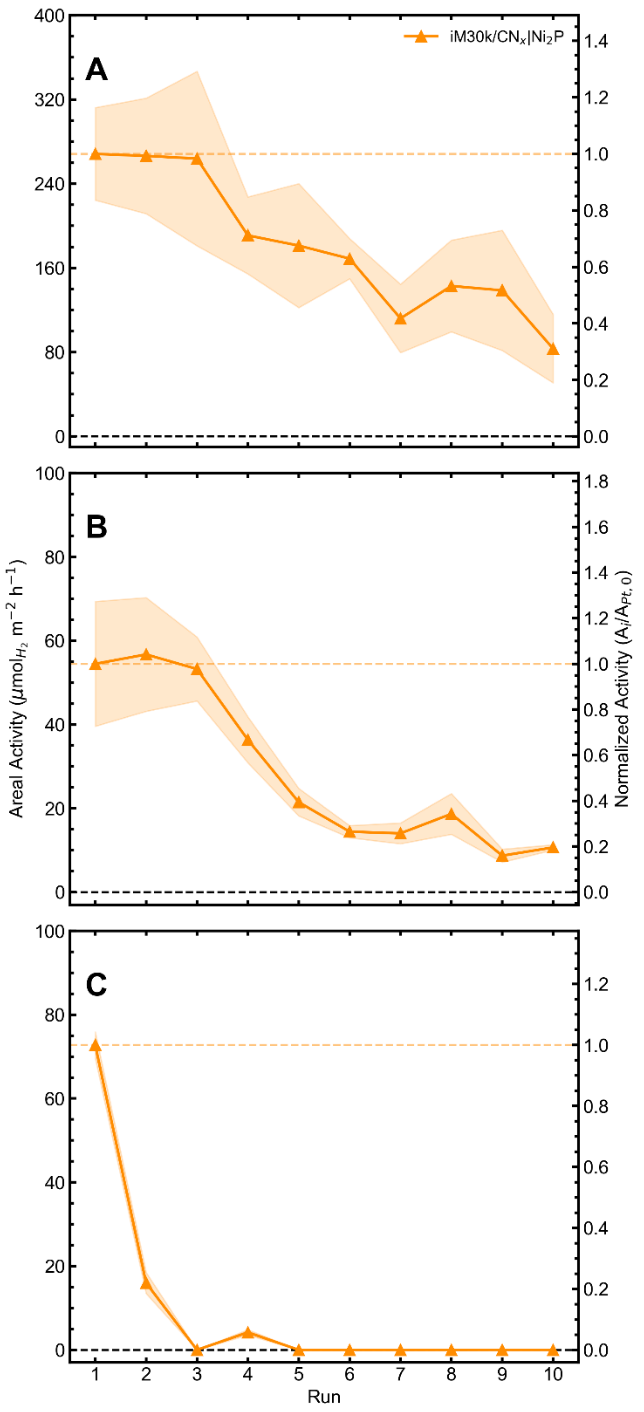

Figure S11. Small area (SA = 4.9 cm^2) solar reforming areal activity of HGM/CN_x|Ni_2P composites using top-down irradiation (100 mW cm^{-2}, V = 5 mL, 1 mol L^{-1} KOH, [substrate] = 25 mg mL^{-1}, no stirring, 2h) over 10 consecutive trials with different substrates: **A** Ethylene glycol, **B** Polyethylene terephthalate, **C** Cellulose. The shaded area surrounding each line shows the standard deviation from each triplicate.

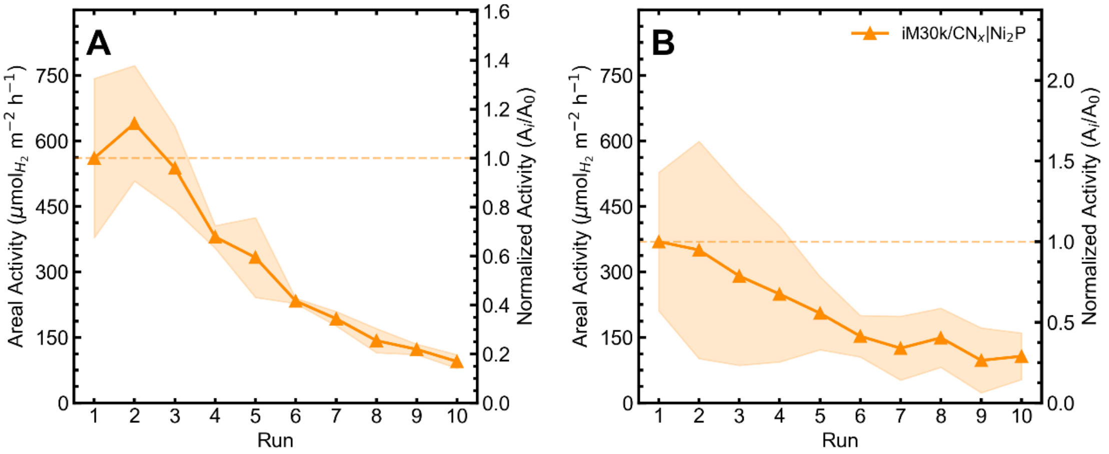

Figure S12. Large area (SA = 217.4 cm^2) solar reforming areal activity of HGM/CN_x|Ni_2P composites using top-down irradiation over 10 consecutive trials (100 mW cm^{-2}, 1 mol L^{-1} KOH, [substrate] = 25 mg mL^{-1}, V = 217 mL, depth = 1 cm, no stirring, 2h, T ≈ 45 °C) with different substrates: **A** Ethylene glycol, **B** Polyethylene terephthalate. The shaded area surrounding each line shows the standard deviation from each triplicate.

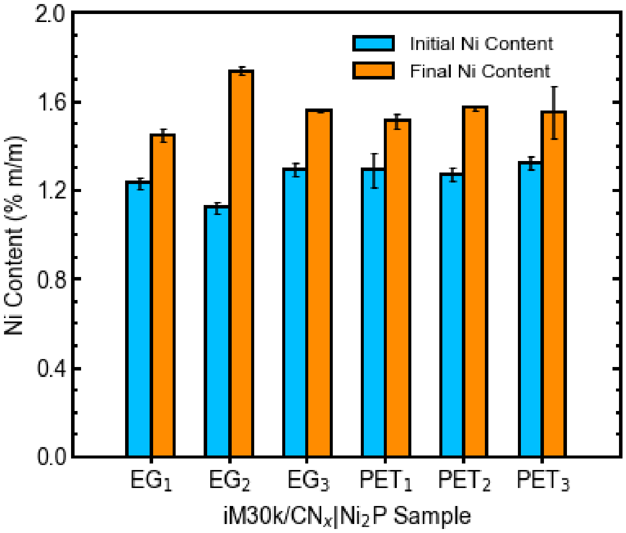

Figure S13. Ni content of iM30k/CN_x|Ni_2P composite measured by ICP-OES before and after 10 consecutive large area (SA = 217.4 cm^2) solar reforming trials using EG or PET substrate. $EG_{1\text{-}3}$ and $PET_{1\text{-}3}$ represent the replicate number for each substrate in the recycling experiment (see Figure 5).

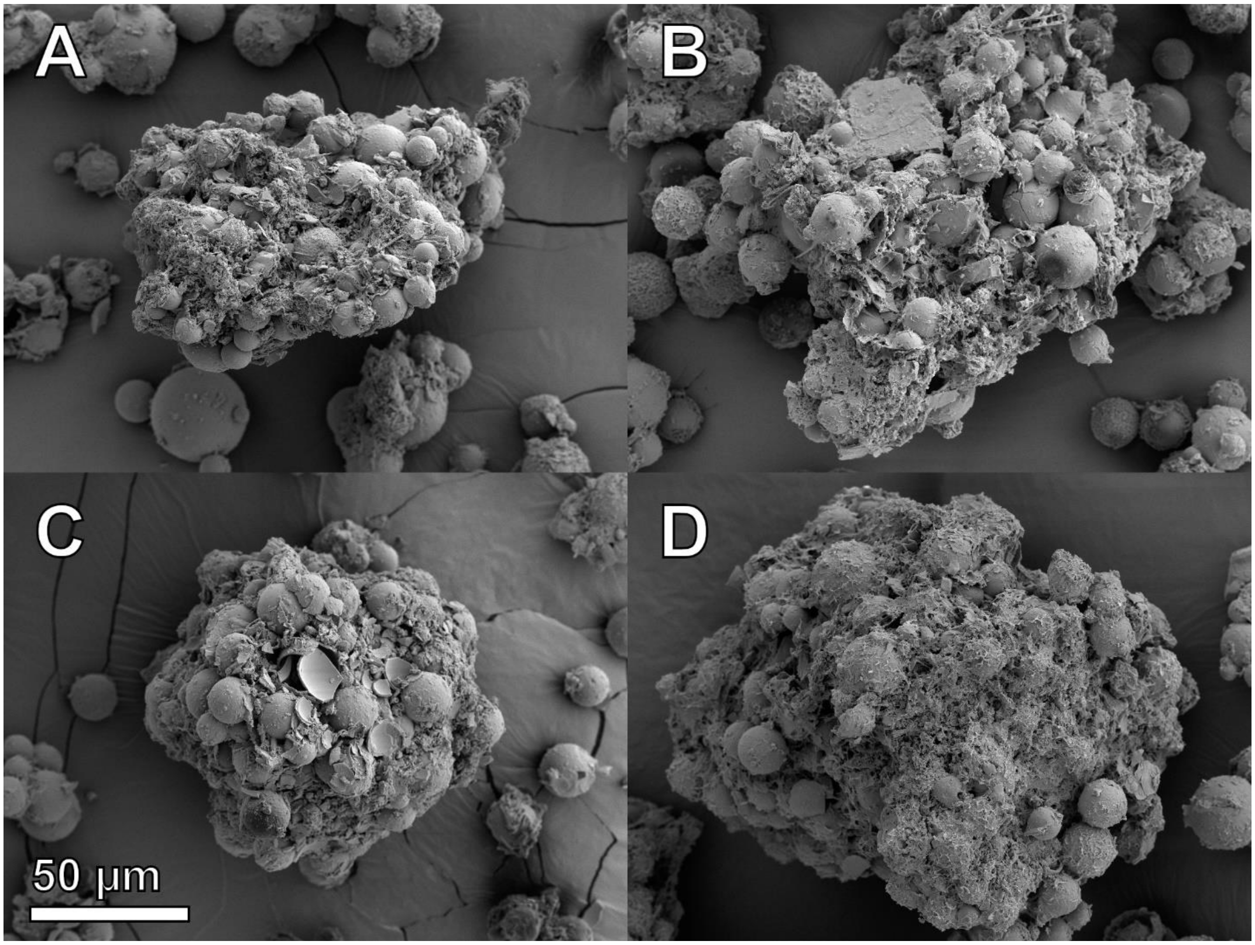

Figure S14. SEM micrographs of the surface of HGM/CN_x|Ni_2P samples before (**A, C**) and after (**B**, **D**) 10 consecutive solar reforming experiments (25 mg mL^{-1} ethylene glycol, 1 mol L^{-1} KOH) in the large reactor. Scale bar is consistent for all panels.

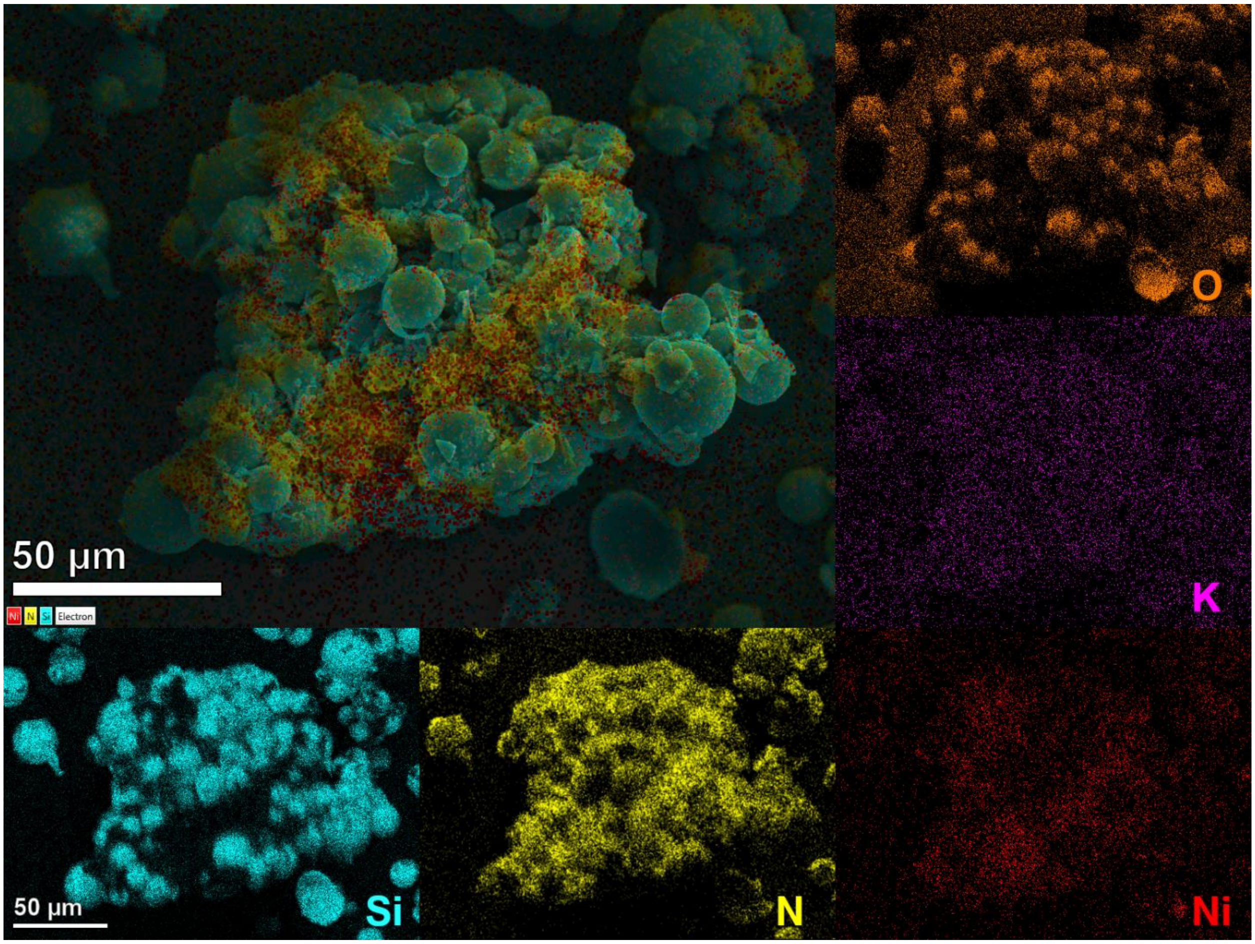

Figure S15. SEM/EDX of HGM/CN_x|Ni_2P composite before using in consecutive solar reforming experiments. The large overlay panel in the top left shows the distribution of Si (teal), N (yellow), and Ni (red) on top of the secondary electron image, while the smaller panels show the individual mapped element signals (Si, N, Ni, K, and O).

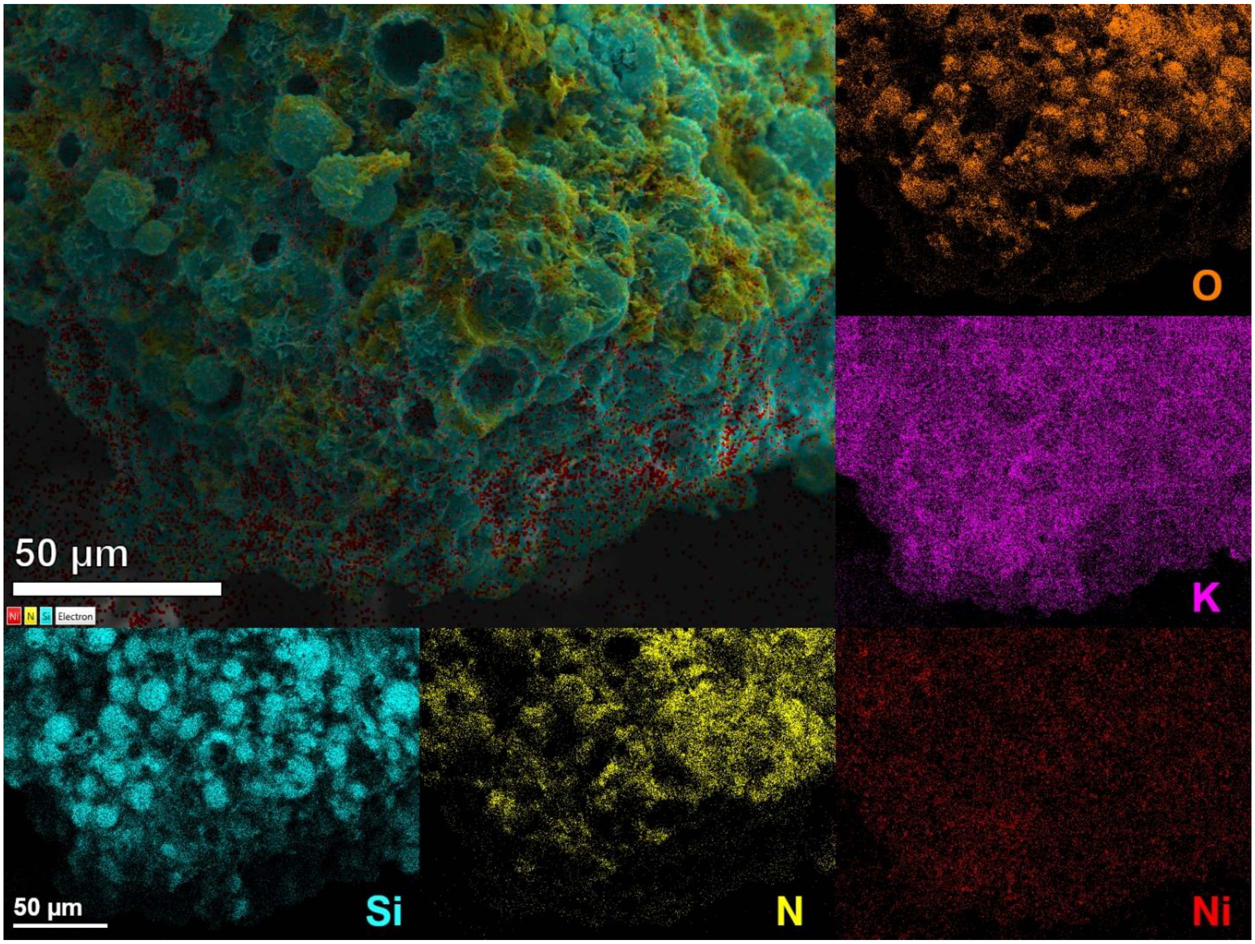

Figure S16. SEM/EDX of HGM/CN_x|Ni_2P composite after 10 consecutive solar reforming experiments (25 mg mL^{-1} ethylene glycol, 1 mol L^{-1} KOH). The large overlay panel in the top left shows the distribution of Si (teal), N (yellow), and Ni (red) on top of the secondary electron image, while the smaller panels show the individual mapped element signals (Si, N, Ni, K, and O).

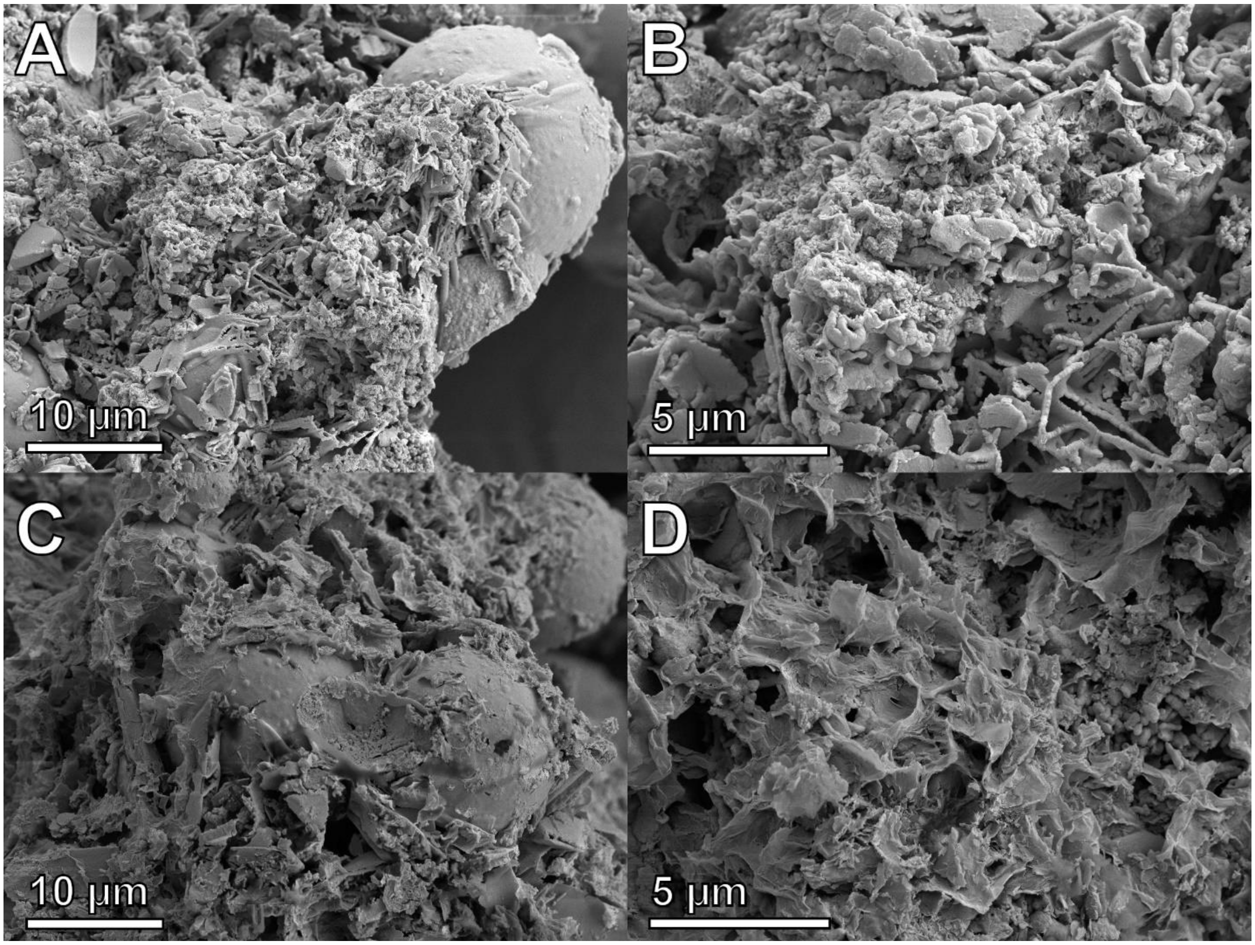

Figure S17. SEM micrographs of the surface of HGM/CN_x|Ni_2P samples before (**A, B**) and after (**C**, **D**) 10 consecutive solar reforming experiments (25 mg mL^{-1} ethylene glycol, 1 mol L^{-1} KOH) in the large reactor.

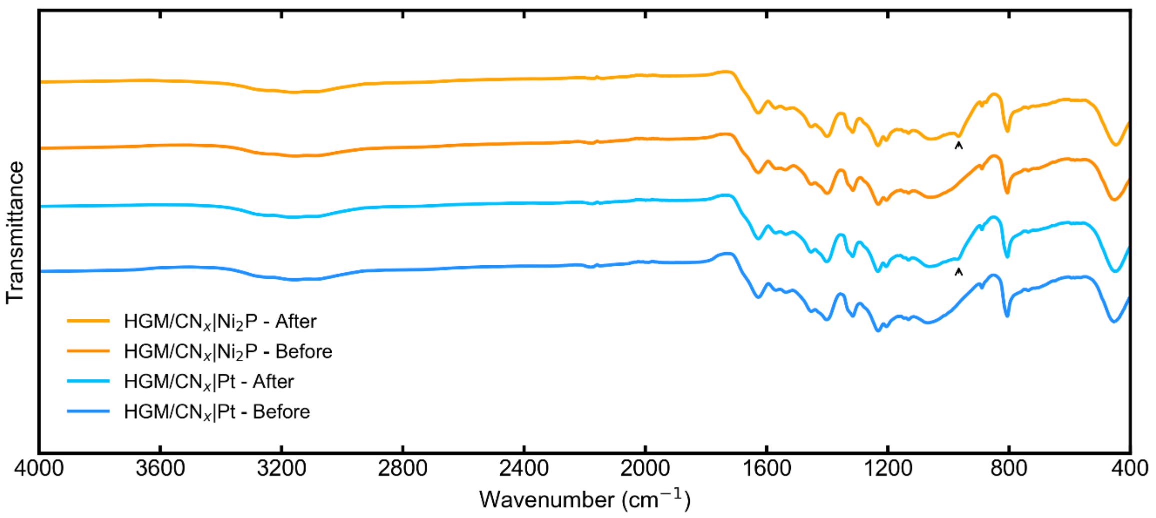

Figure S18. ATR-FTIR of HGM/CN_x composites before and after 10 reuse cycles. The arrows show the appearance of a new peak at 970 cm^{-1} in the 'after' samples.

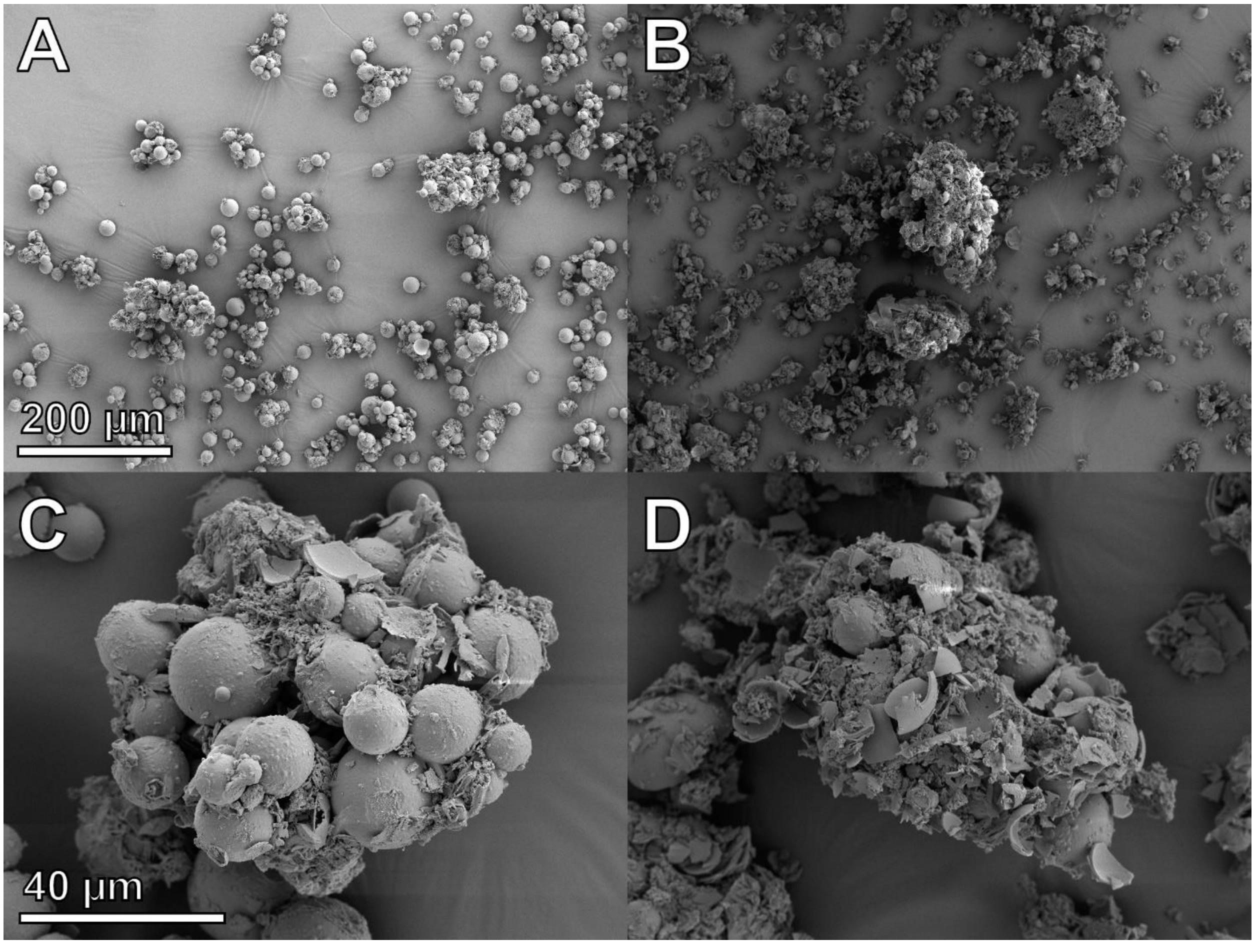

Figure S19. SEM micrographs of HGM/CN_x samples from the floating (**A**, **C**) and sinking (**B**, **D**) fractions after 60 minutes of stirring (100 mg composite in 20 mL water, 50 mL beaker, 2 cm stir bar, 500 rpm). The scale bar in A is consistent for B; the scale bar in C is consistent for D.

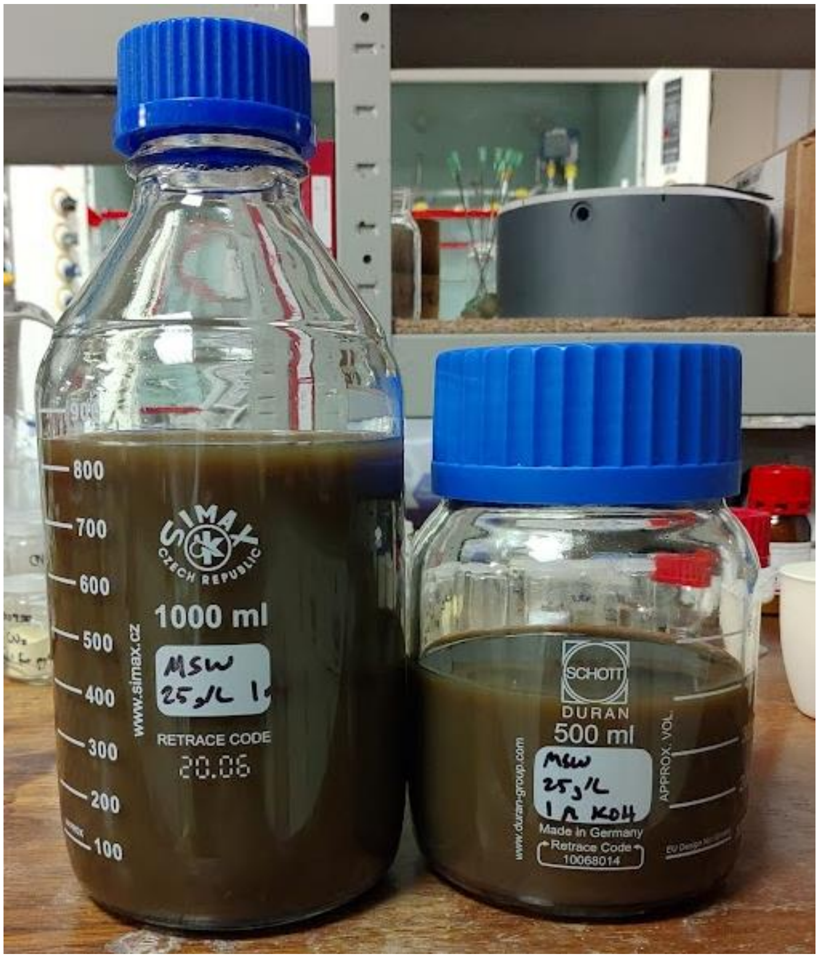

Figure S20. Photograph of turbid waste solution prepared from pre-treated mixed waste (1 mol L^{-1} KOH, 25 g L^{-1} waste, 80 °C, 24 h). 25 mg mL^{-1} EG was added to this suspension for use in the vertically-irradiated SR trials using floating and non-floating CN_x|Pt.

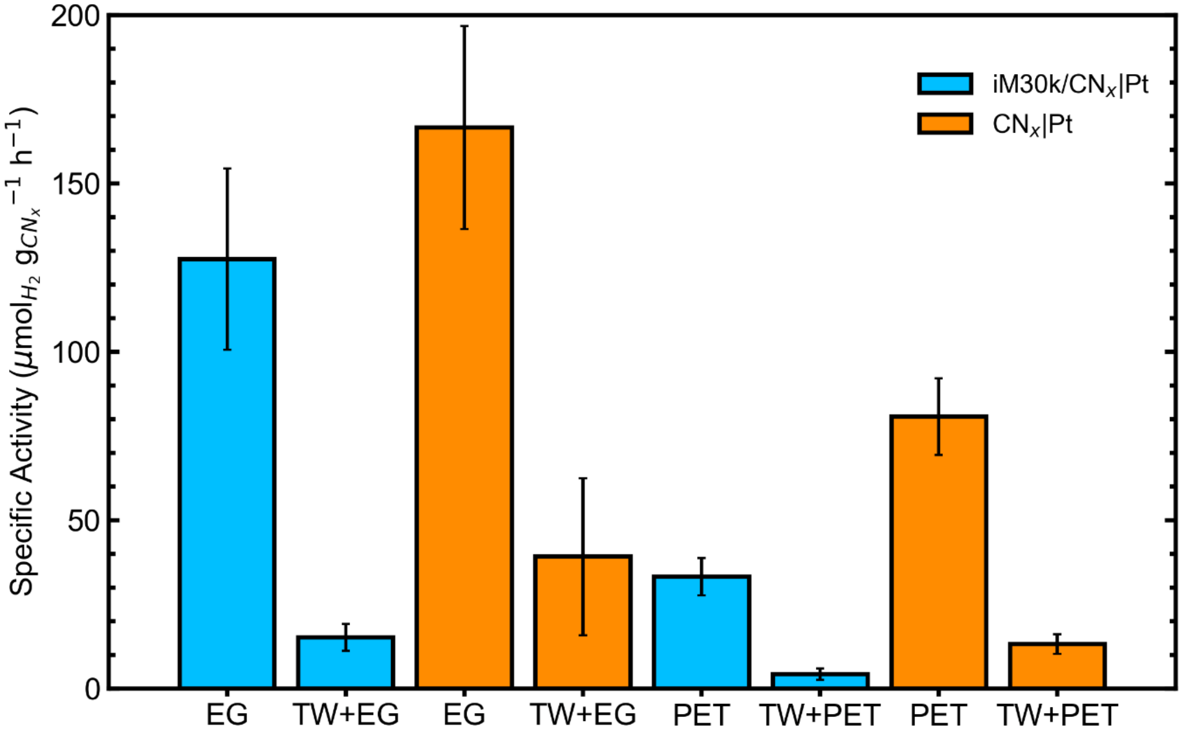

Figure S21. iM30k/CN$_x$ solar reforming using small vials with side irradiation containing transparent ethylene glycol or PET solution (EG, PET) or turbid waste + ethylene glycol or PET solution (TW+EG, TW+PET) (100 mW cm^{-2}, 1 mol L^{-1} KOH, [substrate] = 25 mg mL^{-1}, V = 2 mL, stirred, T = 25 °C). *n.d.* indicates 'not detected'.

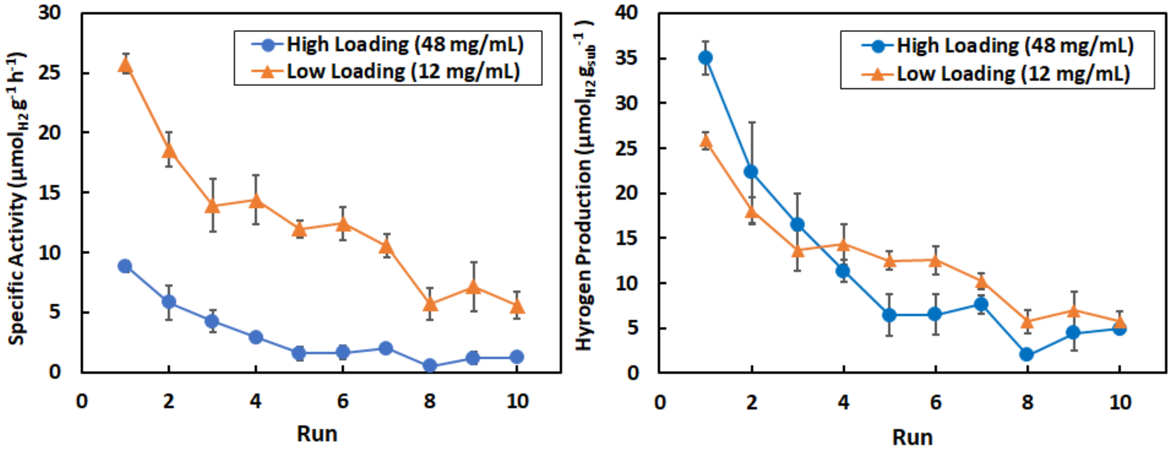

Figure S22. Activity (left panel; μmol$_{H_2}$ g$_{comp}$$^{-2}$ h^{-1}) and yield (right panel; μmol$_{H_2}$ g$_{EG}$$^{-2}$) of floating TiO_2 composite over 10 consecutive solar reforming cycles in a vertically illuminated small reactor (4.9 cm^2, ~46 mL total volume, 5 mL substrate solution volume, 25 mg mL^{-1} EG, 8 μL H_2PtCl_6 (8 % wt.) solution, 2 h exposure periods).

Supporting References

[1] D. S. Achilleos, W. Yang, H. Kasap, A. Savateev, Y. Markushyna, J. R. Durrant, E. Reisner, *Angew. Chem. Int. Ed.* **2020**, *59*, 18184.
[2] X. Brace, E. Matijević, *Colloid & Polymer Sci* **1977**, *255*, 153.
[3] M. A. Gross, A. Reynal, J. R. Durrant, E. Reisner, *J. Am. Chem. Soc.* **2014**, *136*, 356.

End of Supporting Information
